# Integrated multi-omics analysis and machine learning identify G protein-coupled receptor-related signatures for diagnosis and clinical benefits in soft tissue sarcoma

**DOI:** 10.3389/fimmu.2025.1561227

**Published:** 2025-07-21

**Authors:** Duo Wang, Jihao Tu, Jianfeng Liu, Yuting Piao, Yiming Zhao, Ying Xiong, Jianing Wang, Xiaotian Zheng, Bin Liu

**Affiliations:** ^1^ Department of Hand and Foot Surgery, Orthopedics Center, The First Hospital of Jilin University, Jilin University, Changchun, China; ^2^ Engineering Laboratory of Tissue Engineering Biomaterials of Jilin Province, Jilin University, Changchun, China; ^3^ Department of Pathology, National University Hospital (NUH), Singapore, Singapore

**Keywords:** soft tissue sarcoma, G protein-coupled receptors, machine learning, tumor microenvironment, personalized therapy

## Abstract

**Background:**

G protein-coupled receptors (GPRs) are associated with tumor development and prognosis. However, there were fewer reports of GPR-related signatures (GPRSs) in soft tissue sarcomas (STSs), and we aim to combine GPR-related genes with cellular landscape to construct diagnostic and prognostic models in STSs.

**Methods:**

Based on AddModuleScore, single-sample gene set enrichment analysis (ssGSEA), differentially expressed genes (DEGs), and weighted gene co-expression network analysis (WGCNA), GPR-related genes (GPRs) were screened at both the single-cell and bulk RNA-seq levels based on The Cancer Genome Atlas (TCGA) and Gene Expression Omnibus (GEO) databases. We developed a novel machine learning framework that incorporated 12 machine learning algorithms and their 127 combinations to construct a consensus GPRS to screen biomarkers with diagnostic significance and clinical translation, which was assessed by the internal and external validation datasets. Moreover, the GPR-TME classifier as the prognosis model was constructed and further performed for immune infiltration, functional enrichment, somatic mutation, immunotherapy response prediction, and scRNA-seq analyses.

**Results:**

We identified 151 GPR-related genes at both the single-cell and bulk transcriptome levels, and identified a Stepglm[both]+Enet[alpha=0.6] model with seven GPR-related genes as the final diagnostic predictive model with high accuracy and translational relevance using a 127-combination machine learning computational framework, and the GPR-integrated diagnosis nomogram provided a quantitative tool in clinical practice. Moreover, we identified seven prognosis GPRs and five prognosis-good immune cells constructing the GPR score and TME score, respectively. The findings indicate that high expression of GPRs is associated with a poor prognosis in patients with STS, highlighting the significant role of GPRs and the tumor microenvironment (TME) in STS development. Building up a GPR-TME classifier, low GPR combined with high TME exhibited the most favorable prognosis and immunotherapeutic efficacy, which was further performed for immune infiltration, functional enrichment, somatic mutation, immunotherapy response prediction, and scRNA-seq analyses.

**Conclusions:**

Our study constructed a GPRS that can serve as a promising tool for diagnosis and prognosis prediction, targeted prevention, and personalized medicine in STS.

## 1 Introduction

Soft tissue sarcomas (STSs) are a group of the rarest and most extremely heterogeneous malignancies arising from mesenchymal cells, accounting for approximately 1% of all adult malignancies and have a predilection in middle-aged and older adults (1, 2). There are more than 100 different histological and molecular subtypes of STSs and over 50% of patients may experience recurrence and metastasis after surgery. Because of its rarity, its heterogeneous and histological nature, late diagnosis and early metastasis, and the limited responsiveness to chemotherapy, surgery remains the standard treatment and management options have remained unchanged in STS (3, 4). Given that immune checkpoint inhibitor (ICI) agents are widely investigated for treating STS, a series of biomarkers linking GPRs and immune features are emerging, although clinical application remains in its early stages and poses challenges (4, 5). Over the past decades, although there are continuing advances in understanding STS tumorigenesis with molecular biology techniques, the specific etiology of STS remains unknown. Multi-omics bioinformatics strategies can help identify early diagnostic and prognostic biomarkers. With growing interest in the molecular profiling for STS, such approaches could assist clinicians in patient stratification and personalized management within the framework of personalized, predictive, and preventive medicine (PPPM) (6, 7). Thus, the identification of novel appropriate biomarkers for early diagnosis and predicting prognosis is desperately needed in personalized treatment regimens.

G protein-coupled receptors (GPRs) are a large superfamily of cell-surface membrane signaling proteins related to G proteins that can be activated by various ligands involved in a variety of biological processes, including cell adhesion and motion, metabolite signaling transduction, and immune responses (8). Aberrant GPR expression and their functions in relation to metabolites have been addressed in the occurrence and development of various cancers, which have become one of the most important drug targets for drug development (9–11). GPRs can control tumor growth, invasion, migration, survival, and metastasis through their aberrant overexpression, mutation, or increased agonist release (3). Recent studies suggested that acid-sensing GPR may mediate lipogenesis in cancer cells, thereby promoting lipid droplet accumulation and enhancing viability under acidic stress; estrogen-mediated GPR signaling played a critical role in gaining malignant phenotypes (11–13). Moreover, numerous GPRs have been identified to be associated with immunological functions and immune infiltration, such as the activation of A2A receptors and lactate receptors, to be involved in the immune escape of cancer cells in tumor niche, and to promote tumor growth and drug resistance (10, 14). This may provide a novel cancer immunotherapy strategy in STS and obtain potential benefits through the inhibition of the related signaling pathway.

Despite the recognized role of GPR dysregulation in cancers, research on their involvement in STS tumorigenesis remains limited. Meanwhile, it is not clear how GPR-related genes affect the prognosis of patients with STS and whether they can predict the response to immunotherapy in such patients. In the light of these gaps, it is necessary to propose a novel GPR-related gene set that can shed light on the prognosis and biological behavior of STS. Furthermore, the tumor microenvironment (TME) has important implications for tumor growth, metastatic spread, and response to therapy (15). We hypothesized that GPRs are significantly associated with the progression and prognosis of STS and could be a potential biomarker predicting immunotherapy response due to their strong correlation with immunity and chemotherapy drugs. Based on multi-omics analysis and integrative machine learning, we aim to construct a GPR-related signature (GPRS) with the potential to guide early diagnosis, prognosis prediction, targeted prevention, and personalized treatment in the context of PPPM.

## 2 Methods

### 2.1 Datasets

**Figure 1:**
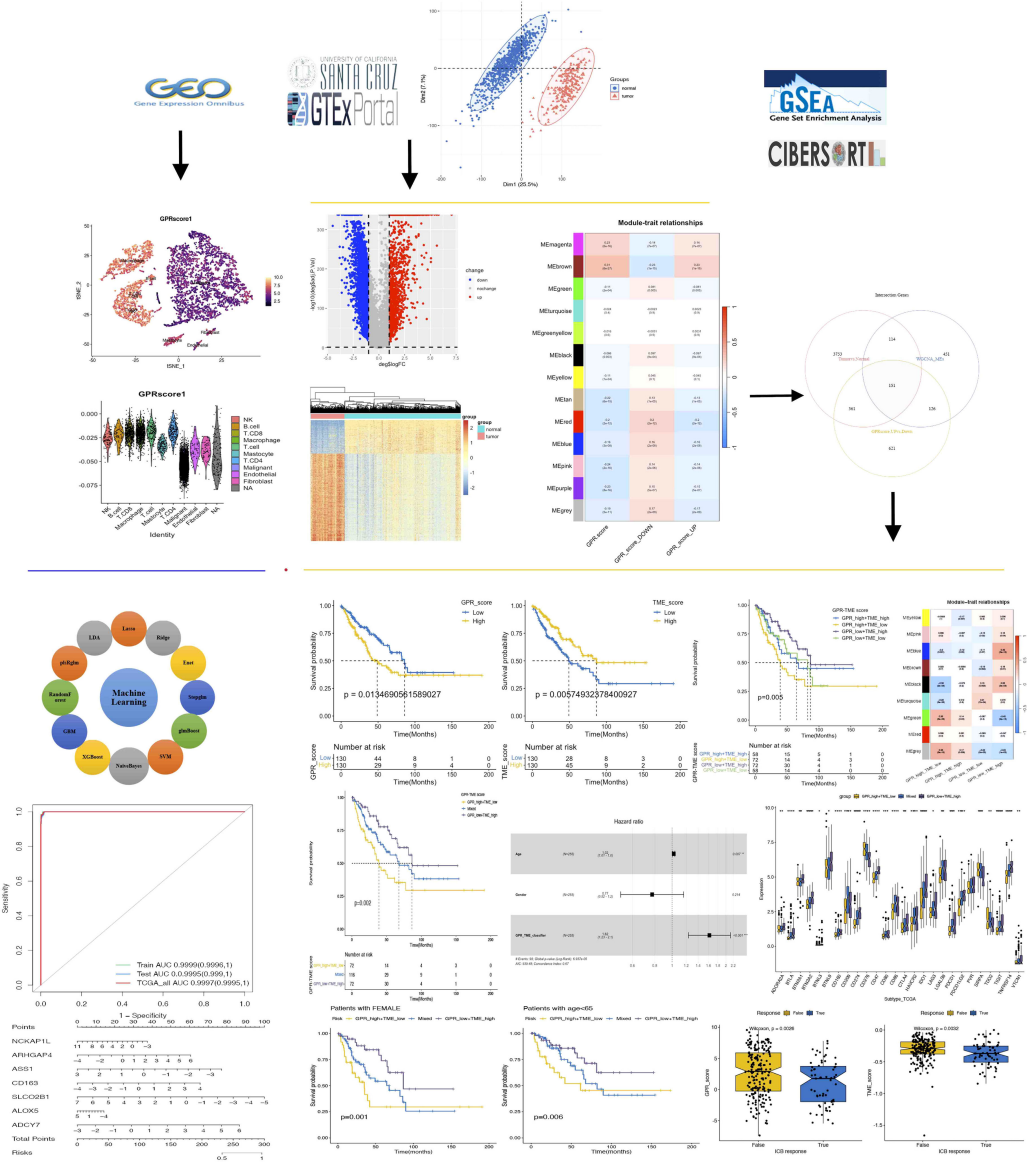
The workflow chart of this study.

The workflow of our study is shown in **Figure 1**. All TCGA-SARC Datasets of transcriptomic, somatic mutations and clinical data for TCGA-SARC came from the UCSC Xena brower. We also obtained the transcriptomic and clinical data of normal adipose and muscle tissues from the Genotype-Tissue Expression (GTEx) database. RNA sequencing (RNA-seq) datasets from these two data portals were processed and unified using the uniform procedures for the direct comparison between tumor and normal tissues at the gene expression level. All gene expression values were TPM-normalized and log2-transformed after the addition of 1, and then used for downstream analysis. We used the *ComBat* function of the *SVA* package to maximize compatibility and reduce batch effects between TCGA and GTEx data. The GSE17674 dataset, consisting of 44 tumor samples and 18 normal controls, was used as an external validation cohort. We also obtained a single-cell RNA sequencing (scRNA-seq) dataset (GSE131309) for sarcomas from the NCBI Gene Expression Omnibus (GEO).

### 2.2 Identification of DEGs and functional enrichment analysis in bulk RNA-seq

We identified differentially expressed genes (DEGs) using the “*limma*” R package with Benjamini–Hochberg method (BH) correction between tumor and normal tissues, and |Log2FoldChange| > 1 and adjusted *p*-values< 0.05 were set as the threshold for screening out significant DEGs. We used the “*clusterProfiler*” R package, and adjusted *p*-values< 0.05 were considered statistically significant for the identification of the related biological functions and signaling pathways of these DEGs, including Gene Ontology (GO) annotation and Kyoto Encyclopedia for Genes and Genomes (KEGG) pathway enrichment analysis, respectively (16).

### 2.3 Single-sample gene set enrichment analysis of G protein-coupled receptors in bulk RNA-seq

The GPR signature used the “G_PROTEIN_COUPLED_RECEPTOR_ACTIVITY” pathway gene lists from MSigDB of the Broad Institute (https://www.gsea-msigdb.org/gsea/msigdb/cards/GOMF_G_PROTEIN_COUPLED_RECEPTOR_ACTIVITY.html), and 870 GPRs were included in further analysis. The single-sample gene set enrichment analysis (ssGSEA) algorithm is commonly used to evaluate changes in biological processes and pathway activity for a single sample. We performed ssGSEA through the R package “GSVA” for obtaining the GPR score for every sample in our study, which reflects the degree to which a specific gene set is systematically changed in the sample by quantifying the enrichment score of a particular gene set within a single sample. Moreover, we classified all samples into up- and down-GPR score groups based on the median ssGSEA score.

### 2.4 Identification of the key module and hub genes to GPRs in bulk RNA-seq

We used the “WGCNA” (weighted gene co-expression network analysis) R package for constructing co-expression networks and identifying gene expression modules based on all RNA-seq data. Pearson correlation coefficients were calculated for all pairwise comparisons of expressed genes across all samples. A soft-threshold power with a scale-free *R^2^* > 0.9 and a slope near −1 was chosen to transform the adjacency matrix to a topological overlap matrix (TOM). Genes with similar expression profiles were classified into modules based on the TOM dissimilarity (1−TOM) with a minimum size of 50 for the gene cluster dendrogram and visualized by hierarchical clustering, and the modules whose eigengenes (MEs) were highly correlated were merged, and high similarity modules were merged to construct the co-expression network. We set the soft-threshold power as 7, cut height as 0.25, and the minimal module size as 50 for module detection and network construction. The module–trait associations were estimated by the correlations of these modules and clinical traits. The modules with the strongest significant correlation with GPR scores via ssGSEA were considered as key modules, and key genes in modules were defined as those with |MM| > 0.8 and |GS| > 0.2. Furthermore, we also performed GO and KEGG pathway enrichment analyses for key modules.

### 2.5 GPRs in single-cell transcriptome by scRNA-seq data analysis

The scRNA-seq (SMART-seq2) dataset (GSE131309) for sarcomas included gene expression profiles from 12 human SyS tumors, and we used the same annotated specific cell clusters from the original research. We used the tSNE algorithm to cluster cell types, which produces a single map to demonstrate structure at many different scales, particularly useful for high-dimensional data. We utilized the “AddModuleScore” and “FindMarkers” functions built in the R “Seurat” package to quantify the activity of the GPR gene set in each cell and analyze the DEGs between high- and low-GPR scores based on the median GPR score in a single cell. The statistical significance of DEGs was determined using Wilcoxon test (*p*_adj_< 0.05), which were considered to be involved in GPRs at the single-cell transcriptome level. Moreover, we also performed cell interaction analysis using the “CellChat” R package.

### 2.6 Identification of G protein-coupled receptor-related signatures

In the TCGA and GTEx bulk RNA-seq and scRNA-seq data, we obtained DEGs and GPR-related modules identified by differential analysis and WGCNA, and DEGs identified by the “FindMarkers” function; these intersected genes were considered to be involved in GPRs at both the bulk and single-cell transcriptome levels, and we referred to them as GPRSs for further analysis.

### 2.7 Construction of a diagnostic model based on integrative machine learning algorithms

To construct a robust diagnostic model with high predictive accuracy, we randomly divided the meta dataset into a training set and an internal validation test set in a 1:1 ratio, ensuring a balanced distribution of clinical characteristics between tumor and normal groups. The meta dataset was used for further internal validation, and GSE17674 was used as an external validation set. Twelve machine learning algorithms were incorporated, including least absolute shrinkage and selection operator (LASSO), Ridge, elastic net (Enet), Stepglm, support vector machines (SVMs), glmBoost, LDA, plsRglm, random forest (RF), generalized boosted regression modeling (GBM), eXtreme gradient boosting (XGBoost), and NaiveBayes. We arranged 127 combinations of these 12 algorithms in the training dataset for variable selection and model construction. All constructed models were evaluated in the internal and external validation dataset, the C index of which was calculated, and then the predictive performance of the models was ranked based on the mean AUROC. We established a final GPRS that can predict the disease with both robust performance and clinically translational significance.

### 2.8 Establishment and validation of a nomogram with GPR-related signatures

To enhance the diagnostic accuracy and predictive ability of our model, we developed a nomogram that incorporated the final seven GPRS genes to quantify the expected patients with STS with the aid of the R “*RMS*” package, and assessed the nomogram’s precision discrimination and accuracy using receiver operating characteristic (ROC) curves, C index, and calibration curves.

### 2.9 GPR-related signatures combined cellular landscape for predicting prognosis and immunotherapy response

#### 2.9.1 Identification of prognostic GPR-related genes and TME cells

To obtain the prognostic-related signatures, we firstly used the R “survival” package to perform univariate Cox proportional regression analysis to screen GPR genes with potential prognostic roles in all datasets and then LASSO Cox regression analysis to further screen gene signatures and reduce overfitting by the R “glmnet” package. Meanwhile, we applied the Kaplan–Meier (K–M) method using the R “survival” and “survminer” packages to screen the protective immune cell types with optimal cutoff analysis, which have a high immune infiltration. We quantified the relative proportions of 22 immune cell types by the “*CIBERSORT*” algorithm with 1,000 iterations and the LM22 gene signature based on the normalization RNA-seq data of tumor and normal tissues. The overall prognostic value of GPR genes and TME cells was presented by hazard ratio (HR) and their 95% confidence interval (CI). Finally, seven GPR genes and five TME cells were identified to be statistically significantly associated with the prognostic outcome in STS (HR< 1 and *p*< 0.05). Correlation analysis was used to study the correlation between the prognostic signatures of seven expression genes and the five infiltrating immune cells via the R “corrplot” package with the default method, which was shown using the heatmap.

#### 2.9.2 Establishment of the GPR score, TME score, and GPR-TME classifier

To ensure the accuracy of the prognostic model, we performed a combination of multivariate Cox regression analysis and the bootstrapping method to construct the GPR score and the TME score, resampling 1,000 times all of the samples using the R “*boot*” package to reduce the overfitting risk and improve the model generalization performance. We obtained the Coef and the bootstrap standard deviation (SD) values of each gene and each cell, and bootstrap–Coef was the ratio of coefficient to SD value of their weight in the corresponding model. The development of the GPR score and TME score was based on the corresponding HR values with bootstrap–Coef values of seven GPR-related genes and five TME cells, respectively (17, 18). The risk scores were calculated using the previous published formula: *Risk score* = $\sum_{i=1}^{n}\frac{Coefi}{SDi}\;\times \;Exp\left(G_{i}\;or\;C_{i}\right)$ , where *G_i_* and *C_i_* were the abundances of the gene or TME cell *i* in each sample. Then, GPR and TME scores were integrated for the development of the GPR-TME classifier, and all tumor samples were further divided into the following subgroups: GPR^low^/TME^high^, intermediate mixed (GPR^low^/TME^low^ and GPR^high^/TME^high^), and GPR^high^/TME^low^ based on the median value of the GPR and TME score in the tumor dataset. The K–M survival of patients with STS was analyzed with the R “survminer” package, and the risk score distribution and survival status were presented. The ROC curves for 3, 5, and 7 years were constructed to evaluate the accuracy of the prognostic model. Furthermore, univariate and multivariate Cox analysis and K–M survival analysis for overall survival (OS) were used to assess the classifier and clinical traits (age and sex) and to identify independent risk factors.

#### 2.9.3 Gene set enrichment analysis and immunological trajectory analysis

We performed gene set enrichment analysis (GSEA) in different GPR score subgroups and TME score subgroups, respectively, using the R “*clusterProfiler*” package with hallmark pathways from “MsigDB” and their DEGs to investigate which hallmark pathways were significantly enriched. Meanwhile, we performed fast gene set enrichment analysis (FGSEA) using the R “*fgsea*” package with hallmark pathways from “MsigDB” to compare the GPR-TME classifier with other gene signatures and their DEGs to investigate which hallmark pathways were significantly enriched. Then, the complex heatmaps of these signatures were built in different GPR/TME subgroups and GPR-TME classifier to compare the consistency and potential pathways. The tracking tumor immunophenotype (TIP) web tool (http://biocc.hrbmu.edu.cn/TIP/) is an online tool that can be used to calculate the activity scores of the seven anti-cancer immune steps for TCGA-SARC samples (19). TIP included seven key steps: release of cancer cell antigens (step 1), cancer antigen presentation (step 2), priming and activation (step 3), trafficking of immune cells to tumors (step 4), infiltration of immune cells into tumors (step 5), recognition of cancer cells by T cells (step 6), and killing of cancer cells (step 7), respectively.

#### 2.9.4 Weighted gene co-expression network analysis based on GPR-TME subgroups

Moreover, we used the “*WGCNA*” R package for constructing co-expression networks and identifying gene expression modules with different GPR/TME subgroups based on all RNA-seq data. We set the soft threshold as 4, the cut height as 0.25, and the minimal module size as 30. The module–trait associations were estimated by the correlations of these modules and different GPR/TME subgroups. The modules with the strongest significant correlation with different GPR/TME subgroups were considered as key modules, and key genes in modules were defined as those with *|*MM| > 0.8 and |GS| > 0.2. Meanwhile, to investigate gene function in each module, we used Metascape web tools (https://metascape.org/gp/index.html) to perform GO enrichment and cluster analysis with the following ontology sources: GO Biological Processes, KEGG pathway, Canonical Pathways, Reactome Gene Sets, DisGeNET, TRRUST, and COVID (20).

#### 2.9.5 Tumor somatic mutation, immunotherapy response, and Proteomaps analysis

Somatic mutation data of TCGA-SARC were available in the TCGA-SARC database. The top 20 mutation genes were obtained and then compared between GPR-TME subgroups. Oncoprints for these genes were built by the R package “ComplexHeatmap.” Candidate genes with significant differences among GPR-TME subgroups were then extracted for further prognosis analysis. The tumor mutational burden (TMB) score of each tumor was also calculated using previously described methods. We collected immune checkpoint-related genes and HLA class genes from the literature. Subsequently, we analyzed the expression differences of individual immune checkpoint genes and HLA class genes between the high-risk group and the low-risk group, with *p*< 0.05 as the standard. Tumor immune dysfunction and exclusion (TIDE) (http://tide.dfci.harvrd.edu/) is an online tool that can be used to calculate the response score of immunotherapy (21). In order to explore the differences in the immunotherapy response of GPR^low^/TME^high^ and GPR^high^/TME^low^ groups, we standardized the TCGA-SARC dataset expression profile using the R package “scale” and calculated the TIDE. We obtained DEGs between GPR^low^/TME^high^ and GPR^high^/TME^low^ groups for Proteomaps analysis, which were developed by a web tool (https://bionic-vis.biologie.uni-greifswald.de/) (22). Finally, the association of the GPR-TME classifier with immunotherapy was explored by comparing the similarity of upregulated genes as well as downregulated genes in both groups.

### 2.10 Statistical analysis

All statistical analyses and data visualization were performed using the R 4.1.3 software. DEGs between groups were identified by using the R “limma” package and FDR-corrected *p*-value to assess the significant differences in DEGs. K–M survival analysis and the log-rank test were performed to compare the OS among different subgroups using the R “survival” and “survminer” packages. Univariate and multivariate Cox regression analyses were used to screen prognostic signatures and construct risk scores. The log-rank test and Cox proportional hazard regression were used to investigate independent prognostic factors. ROC curve analysis and calculation of the area under the curve (AUC) were performed using R “timeROC”. Correlation analysis between potential prognostic genes and immune cells was performed using Spearman’s correlation test. The differences between two non-normally distributed variables were estimated by the Wilcoxon test as a non-parametric method. All statistical tests were two-sided, and *p*< 0.05 was considered statistically significant.

## 3 Results

### 3.1 Identification of DEGs and functional enrichment analysis

**Figure 2:**
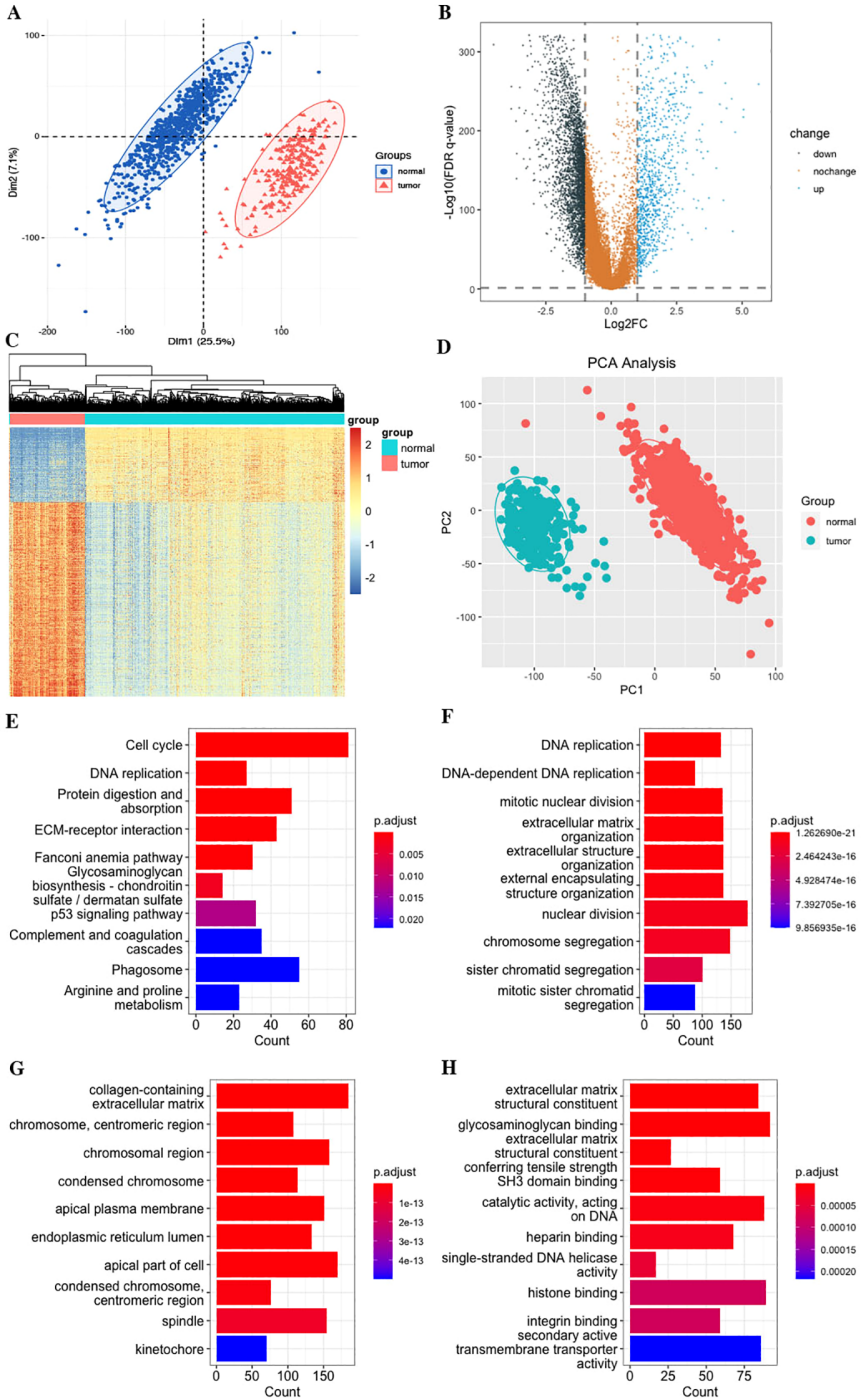
Differentially expressed genes (DEGs) of STS. **(A)** PCA of the combined TCGA and GTEs datasets. **(B)** Volcano plot. **(C)** Heatmap plot. **(D)** PCA plot. **(E)** KEGG pathways. **(F)** Biological process (BP). **(G)** Cellular component (CC). **(H)** Molecular function (MF).

A total of 4,379 significant DEGs were identified by differential expression analyses according to the selection criteria, including 3,166 downregulated and 1,213 upregulated significant DEGs (**Figures 2A–D**). To explore the biological functions of these DEGs, GO and KEGG enrichment analyses were performed. The top 10 KEGG pathways were as follows: Cell cycle, DNA replication, Protein digestion and absorption, ECM–receptor interaction, Fanconi anemia pathway, Glycosaminoglycan biosynthesis, p53 signaling pathway, Complement and coagulation cascades, Phagosome, and Arginine and proline metabolism (**Figure 2E**). The most abundant GO terms were for biological process nuclear division (BP), collagen-containing extracellular matrix (CC), and glycosaminoglycan binding (MF), respectively (**Figures 2F–H**).

### 3.2 Identification of key gene expression modules related to GPR in bulk RNA-seq

**Figure 3:**
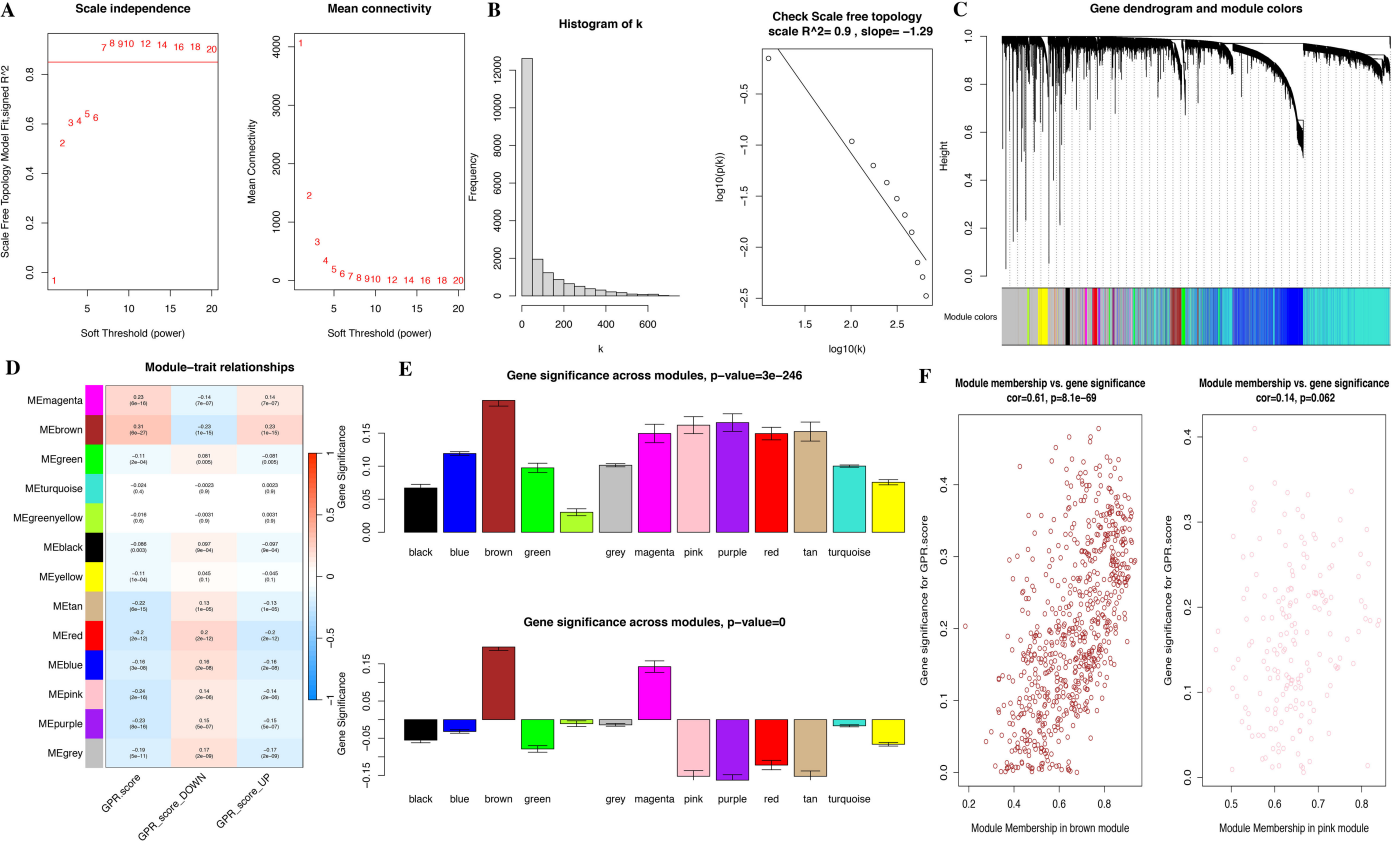
Identification of co-expressed modules and relationship of modules and GPR score by bulk RNA-seq. **(A)** Soft threshold of scale free topology model. **(B)** Histogram. **(C)** Cluster dendrogram of all genes. **(D)** Correlation of modules and GPR score. **(E)** Bar plot of mean gene significance (GS) across modules. **(F)** Scatterplots of GS for disease status versus module membership (MM) in four key modules.

In the study, we performed the ssGSEA algorithm to obtain the GPR activity score for each STS tumor and normal sample, which served as the clinical trait for further WGCNA. To identify key modules associated with the GPR score, we applied WGCNA to the combined TCGA and GTEx datasets to construct the co-expression network. The cluster dendrogram of genes and the module–trait relationship are presented in **Figure 3**. A soft-threshold power value of 7 (*R^2^* = 0.9) was set to ensure a scale-free topological network with high-scale independence and low mean connectivity of all genes. We identified 13 modules ranging in size from 76 genes in the tan module to 7,358 in the turquoise module, with a gray module not belonging to any modules (**Figures 3A–D**). Our findings indicated that the MEbrown module was positively correlated with the GPR score in bulk RNA-seq, indicating that genes in the brown module were mostly overexpressed in GPR score, and pink module genes were negatively correlated with GPR score, meaning that those genes were mostly underexpressed in GPR score (**Supplementary Table S1**). Moreover, the scatterplot of GS versus module membership (MM) shows a significant correlation in the brown module but only a slight correlation in the pink module (**Figure 3E**), indicating that module genes may have functional significance associated with GPR.

**Figure 4:**
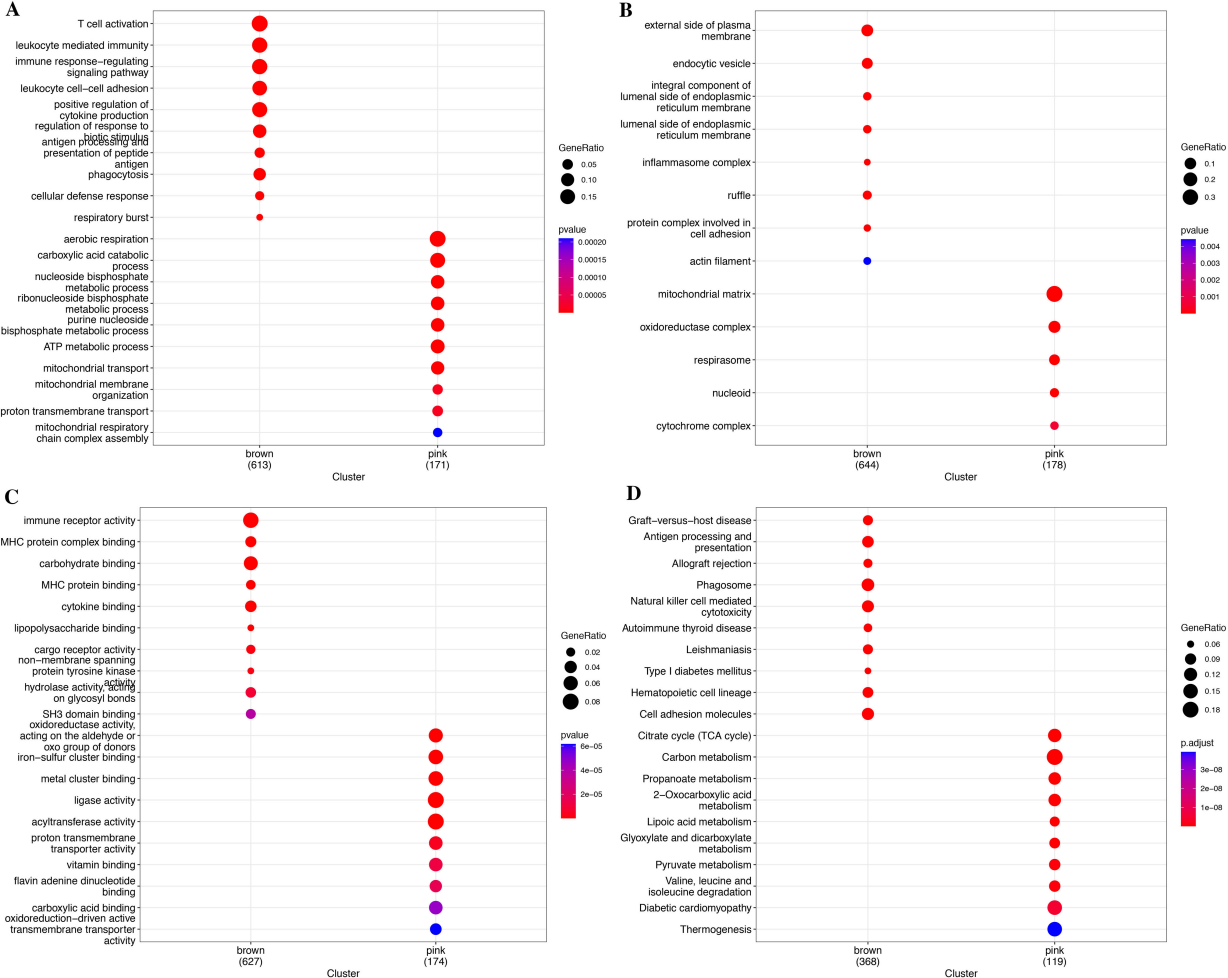
Functional enrichment analysis for module genes. **(A)** KEGG pathways. **(B)** Biological process (BP). **(C)** Cellular component (CC). **(D)** Molecular function (MF).

Meanwhile, we performed functional enrichment analyses in these two key modules since module genes with similar expression patterns might take part in parallel biological procedures or networks. The results of KEGG pathway and GO enrichment analyses of the two key modules are shown in **Figure 4**. KEGG pathway analyses revealed that brown module genes mainly participate in Graft-versus-host disease, Antigen processing and presentation, Allograft rejection, Phagosome, Natural killer cell-mediated cytotoxicity, Autoimmune thyroid disease, Leishmaniasis, Type I diabetes mellitus, Hematopoietic cell lineage, and Cell adhesion molecules; pink module genes mainly participate in Citrate cycle (TCA cycle); Carbon metabolism; Propanoate metabolism; 2-Oxocarboxylic acid metabolism; Lipoic acid metabolism; Glyoxylate and dicarboxylate metabolism; Pyruvate metabolism; Valine, leucine, and isoleucine degradation; Diabetic cardiomyopathy; and Thermogenesis (**Figure 4D**; **Supplementary Table S2**). The most abundant GO terms of brown module genes were T-cell activation, external side of plasma membrane and immune receptor activity for biological process nuclear division (BP), collagen-containing extracellular matrix (CC), and glycosaminoglycan binding (MF), respectively (**Figures 4A–C**; **Supplementary Tables S3**-**S5**). Aerobic respiration, mitochondrial matrix and oxidoreductase activity, acting on the aldehyde or oxo group of donors were the most significant terms in pink module. Furthermore, under the threshold of |MM| > 0.8 and |GS| > 0.2, we identified a total of 150 key genes, including 144 genes in the brown module and 6 genes in the pink module (**Figure 3F**).

### 3.3 G protein-coupled receptor characteristic in the single-cell transcriptome level

**Figure 5:**
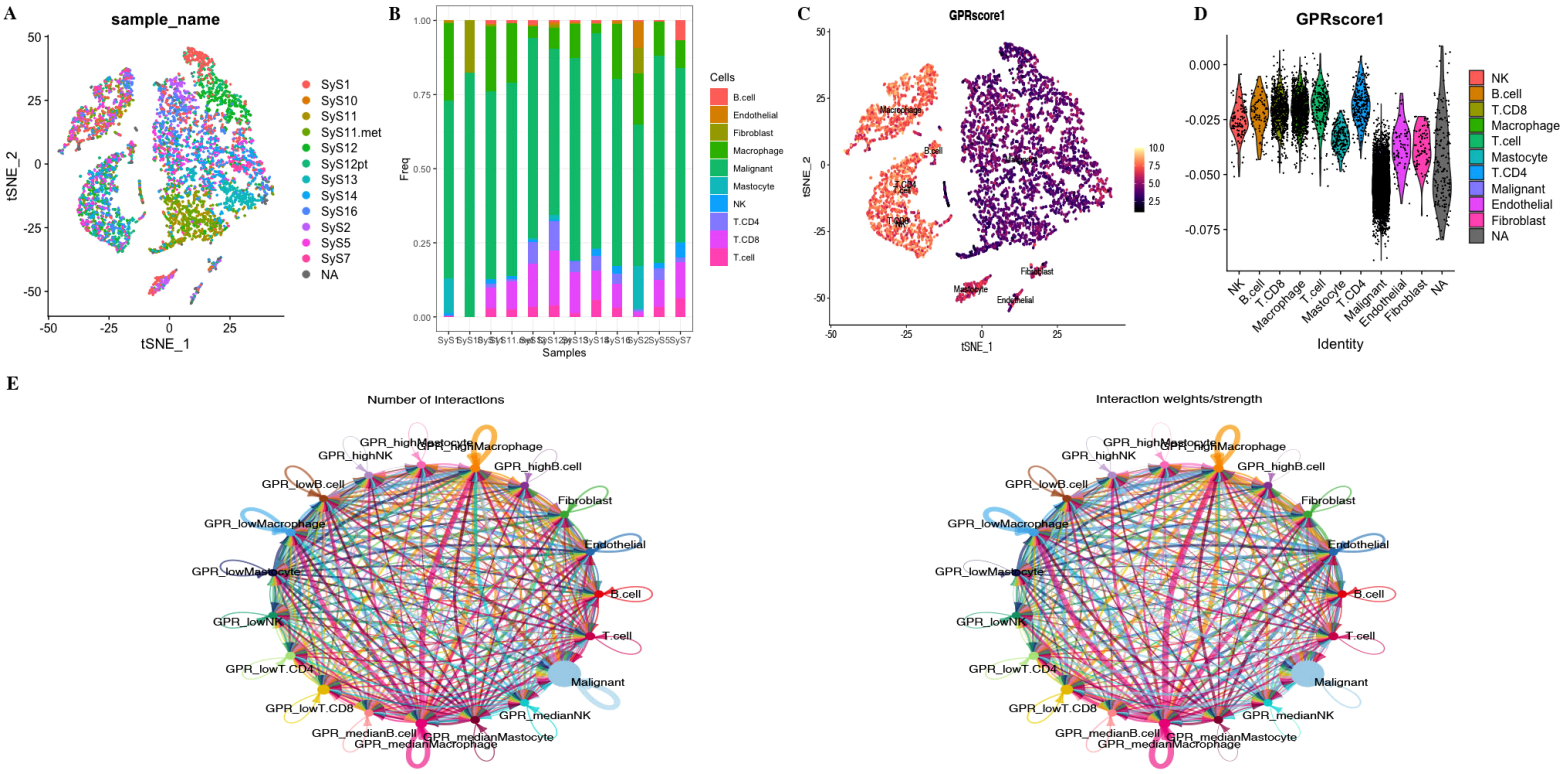
**(A)** tSNE map of cell clusters in STS sample. **(B)** Bar graph shows the proportion of cell types in each sample. **(C, D)** GPR score counted in each cell cluster. **(E)** The number of interactions and the interaction strength of intercellular communication analysis.

We used the annotated cell types in scRNA-seq data, including B cells, endothelial cells, fibroblasts, macrophages, malignant cells, mastocytes, NK cells, CD4+ T cells, CD8+ T cells, and T cells. We used the “AddModuleScore” function in the R “Seurat” package to calculate the expression levels of the GPR gene set across all cells, quantifying the activity of the GPR (GPRScore) in different cell types. Of the 10 cell types, we observed a significantly higher GPR activity in B cells, macrophages, NK cells, CD4+ T cells, CD8+ T cells, and T cells (**Figures 5A–D**). Moreover, we further performed differential analyses based on GRP activity, and we classified all cells into high- and low-GPR groups, and identified 1,259 DEGs between these two groups for further analysis.

**Figure 6:**
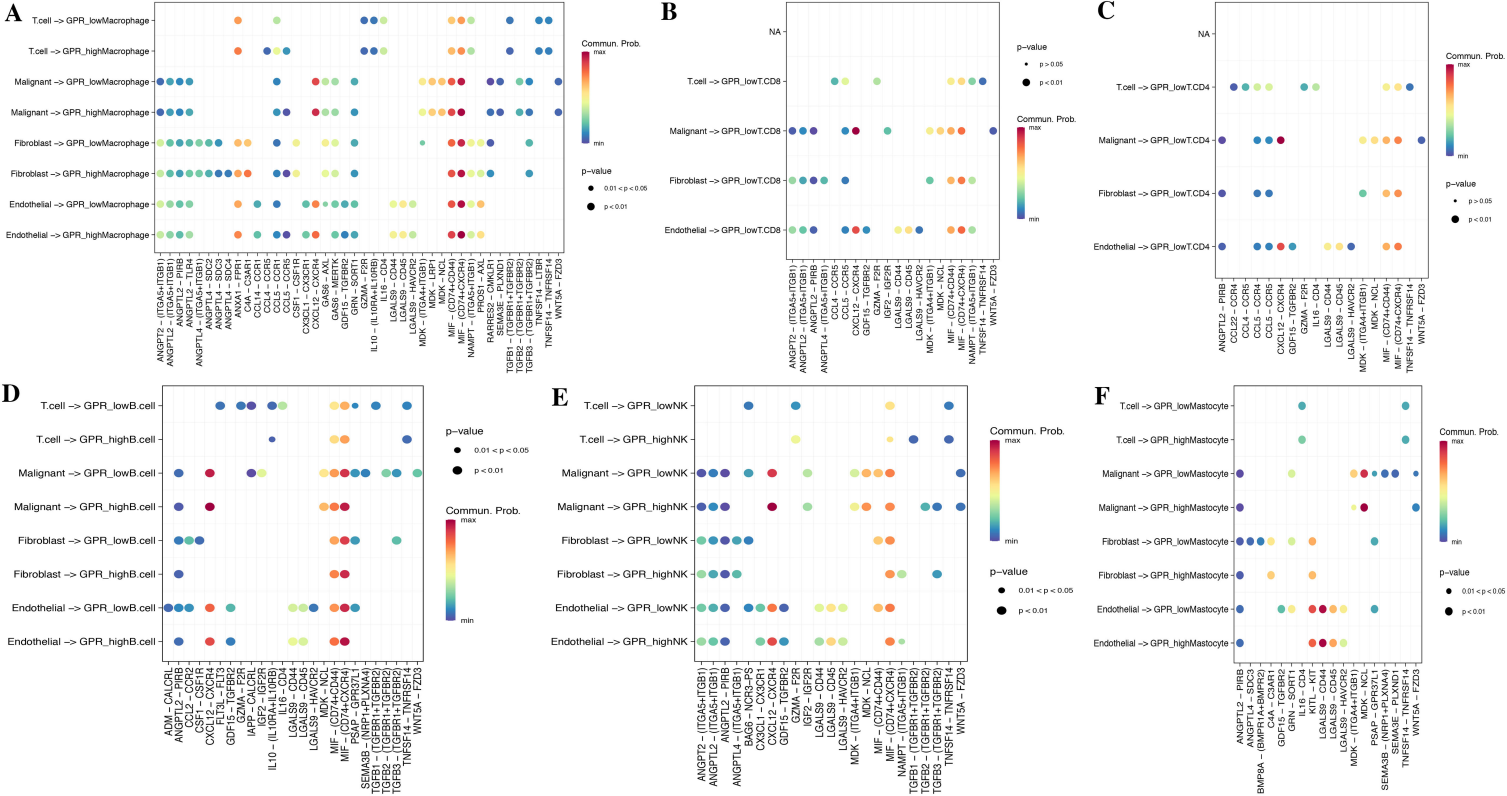
Cell–cell communication analysis. Dot plots of entry and exit interaction signaling pathways in macrophage **(A)**, CD8+ T **(B)**, CD4+ T **(C)**, B cell **(D)**, NK **(E)**, and mastocyte cell **(F)** with high and low GPR activity.

To investigate the role of GPRScore in the TME at the single-cell transcriptome level, we selected those cells to bind to GPR scores and performed cell–cell communication analyses for intercellular communication between various cells. The number of interactions and the interaction weights of the cell–cell communication network are presented in **Figure 5E**. We divided B cells, CD4+ T cells, CD8+ T cells, macrophages, and mastocytes into high- and low-GPR score groups using the quartiles as the boundary, and investigated their interactions with other types of cells in the TME. We found that TME cells with different GPR scores had diverse communication patterns. The results indicate that TME plays a crucial role in cellular communication; those cells with GPR activity possess an additional ability to communicate with different cell types through multiple pathways. We found that B, NK, Macrophage cells and Mastocyte cells in both GPR-high and GPR-low risk groups and CD4+ and CD8+ T cells in GPR-low risk group interacting with MIF-(CD74 + CXCR4) ligand–receptor relationships were extremely correlated, which indicated that the interactions of those cells with other cell types are related to MIF signaling pathway (**Figures 6A–F**). These findings suggest that high GPR activity enhances the cellular communication function of macrophage cells while suppressing the cellular communication function of B cells, CD4 + T cells, CD8 + T cells, NK cells, and mastocyte cells.

### 3.4 Identification of GPR-related genes

**Figure 7:**
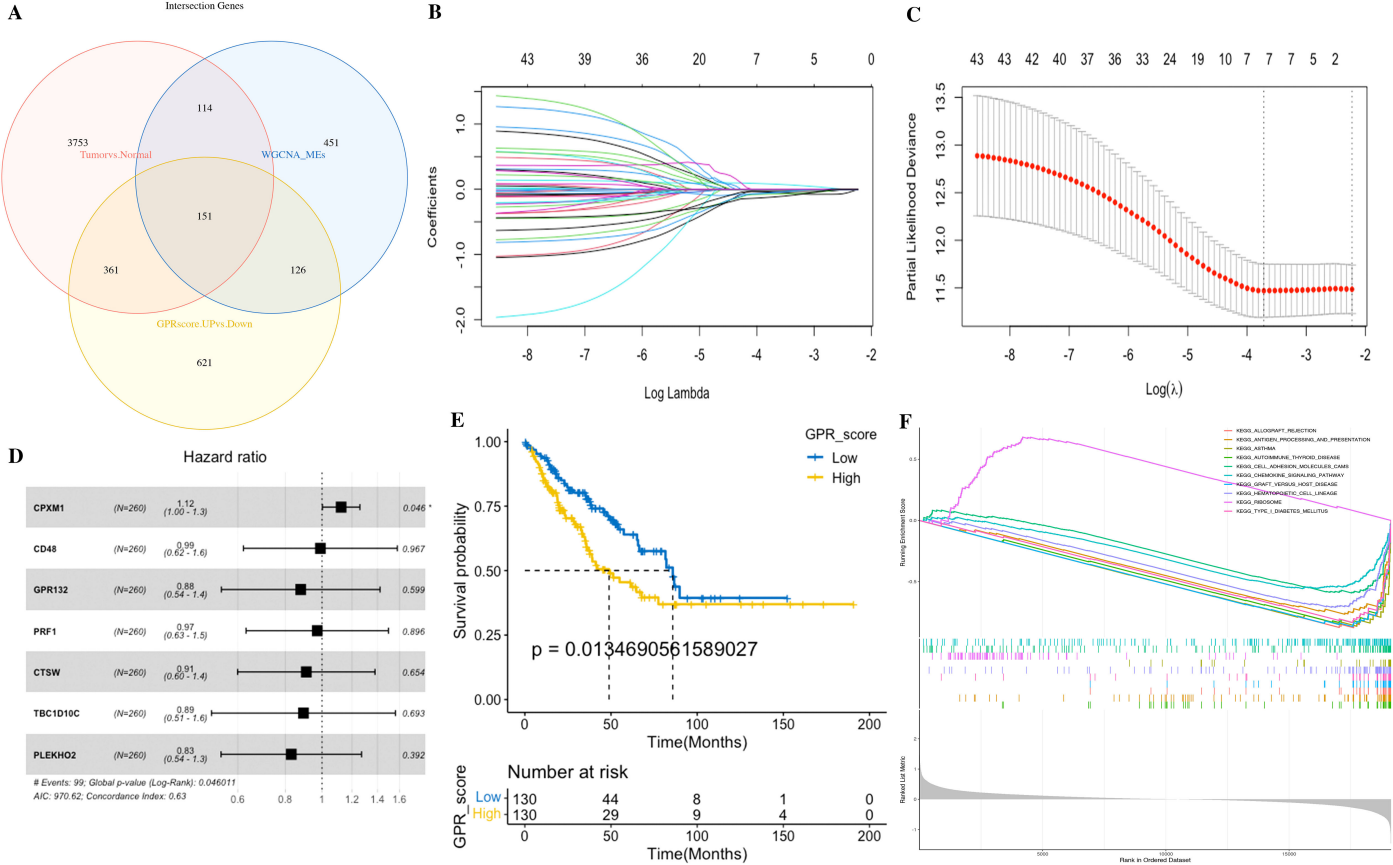
Identification of the GPR-related genes (GPRgenes) and development of the GPR score in STS. **(A)** Venn plot showing the intersecting genes, DEGs, and key module genes from the bulk RNA-seq and the DEGs from the scRNA-seq. **(B)** Selection of the optimal λ in the LASSO analysis. **(C)** LASSO coefficient profiles of genes in STS. **(D)** Forest plot shows a multivariate Cox analysis of these enrolled genes. **(E)** K–M curves for the OS of patients with STS in the low- and high-risk subgroups based on the GPR score. **(F)** Gene set enrichment analysis (GSEA) in the low- and high-risk subgroups based on the GPR score.

Based on GRP activity, we classified all cells into high- and low-GPR groups, and identified 1,259 DEGs between these two groups for further analysis. We intersected the DEGs, key module genes from the bulk RNA-seq, and the DEGs by the “FindMarkers” function from the scRNA-seq, finally identifying a total of 151 genes for further analysis (**Figure 7A**; **Supplementary Table S6**). These genes were named GPR-related genes (GPR genes), which were considered to be involved in GPR at both the bulk and single-cell transcriptome levels.

### 3.5 Establishment and validation of the diagnostic risk prediction model

**Figure 8:**
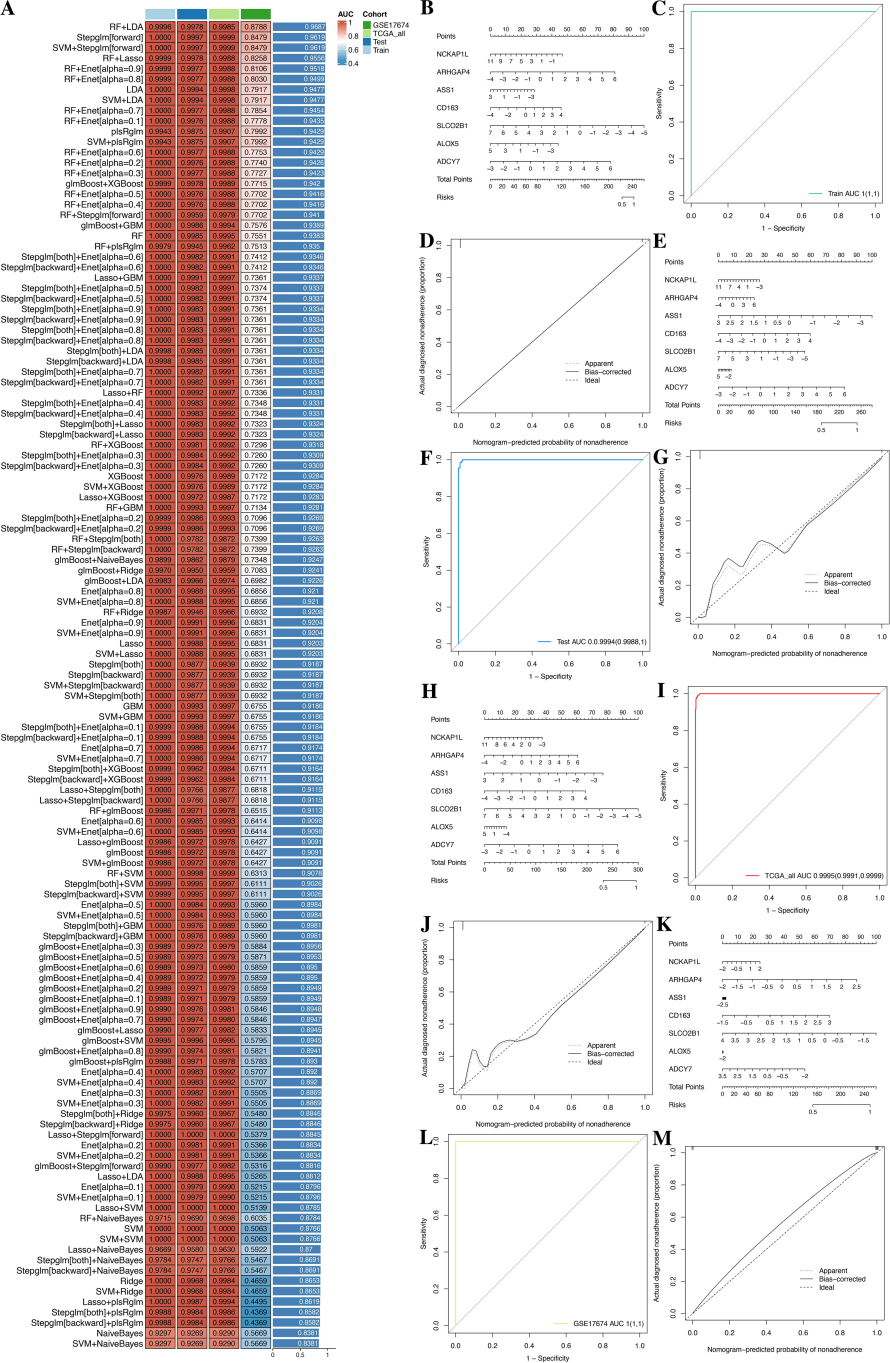
A consensus GPRS was developed and validated via the machine learning-based integrative procedure. **(A)** A total of 127 kinds of prediction model frameworks; the C index of each model was further calculated across all datasets. **(B–D)** Establishment, ROC, and calibration curve of the nomogram in the train, test, meta, and GSE17674 sets. **(E–G)** Establishment, ROC, and calibration curve of the nomogram in the test set. **(H–J)** Establishment, ROC, and calibration curve of the nomogram in the meta set. **(K–M)** Establishment, ROC, and calibration curve of the nomogram in GSE17674.

To construct a consensus GPRS, we performed a combination of 127 machine learning algorithms to analyze the 151 GPR genes; the combined dataset was divided into a training set and an internal test set at a 1:1 ratio, and the meta set and GSE17674 were used for further validation. In the training set, we fitted 127 diagnostic prediction models and calculated the AUROCs across all training and validation datasets (**Figure 8A**). We identified that the Stepglm[both]+Enet[alpha=0.6] model presented, which demonstrated good predictive ability in the training, test, and meta datasets, only incorporated seven genes (NCKAP1L, ARHGAP4, ASS1, CD163, SLCO2B1, ALOX5, and ADCY7), yet achieved comparable predictive efficacy with high accuracy and translational relevance (**Supplementary Tables S7**, **S8**). Moreover, ROC curves showed that the Stepglm[both]+Enet[alpha=0.6] model had a good diagnostic effect (**Figure 8A**; **Supplementary Table S7**). Therefore, these genes may have the potential for early diagnosis. In order to improve the clinical applicability of the diagnostic model, we constructed a nomogram based on these genes and also plotted the calibration curves. The C indexes of the nomogram were 1, 0.9994, 0.995, and 1 in the train, test, meta, and GSE17674 sets (**Figures 8B–M**). The calibration chart predicted by the nomogram was in excellent consistency with the actual observation results in the training, test, meta, and GSE17674 sets. The above confirms that those genes might have the potential as diagnostic markers.

### 3.6 Establishment of a prognostic risk model for the combined signatures of GPRs and cellular landscape

Subsequently, we performed univariate Cox regression analysis on the 151 GPR genes, identifying 44 significant genes (*p*< 0.05). Then, to reduce overfitting, we conducted LASSO Cox regression analysis in the tumor set, yielding seven potential prognostic genes (CPXM1, CD48, GPR132, PRF1, CTSW, TBC1D10C, and PLEKHO2 in **Supplementary Table S9**). Therefore, we performed the multivariate Cox regression analysis with bootstrap methods based on these seven genes to establish a prognostic model, resampling 1,000 times with a multivariate Cox analysis each time, and the ratio of Coef to bootstrap SD values serves as the weight in the corresponding model for improving the stability of the prognostic model (**Figures 7B–D**).

**Figure 9:**
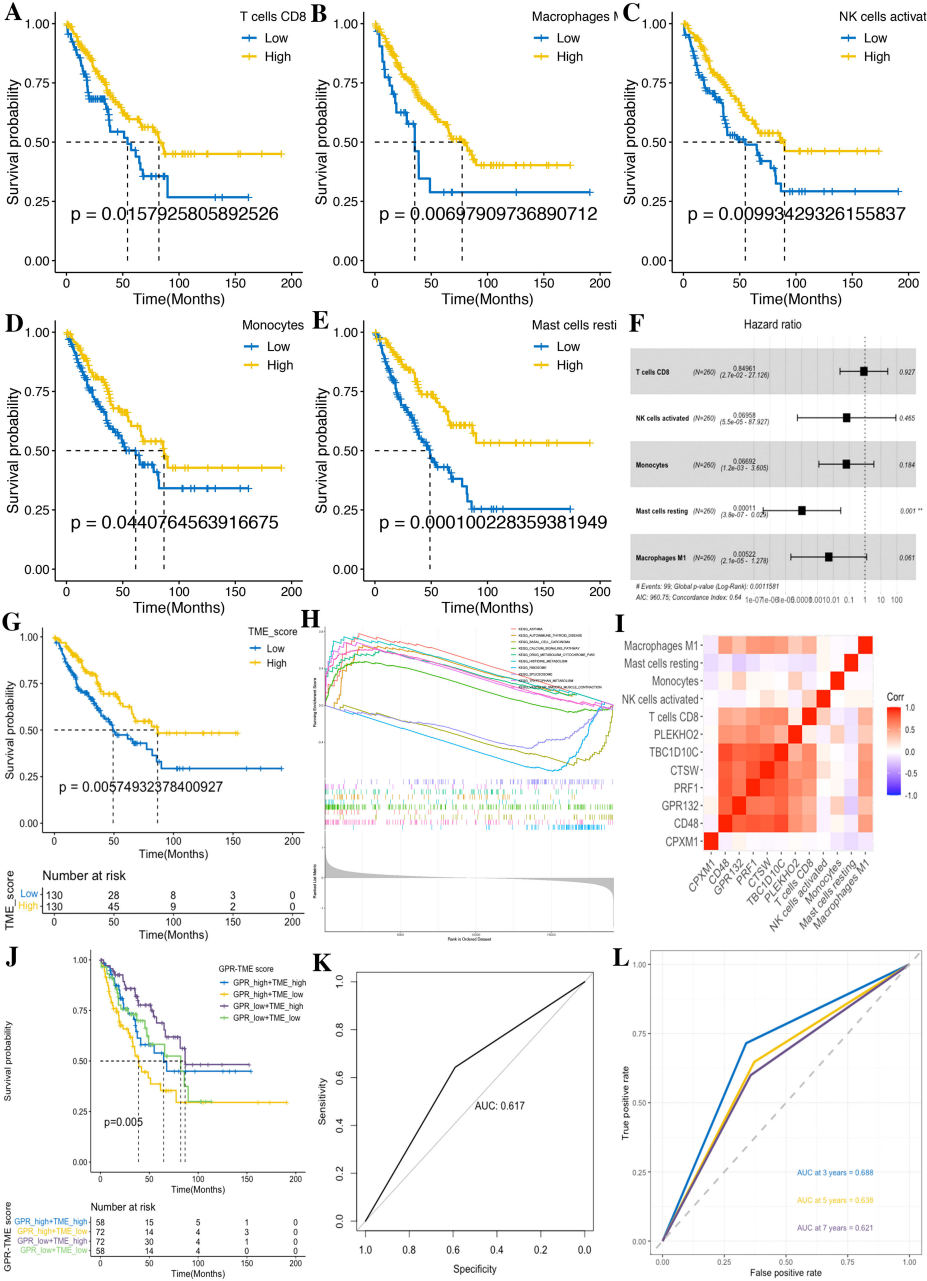
Development of the TME score in STS. **(A)** CD8 T cells. **(B)** M1 macrophages. **(C)** Activated NK cells. **(D)** Monocytes. **(E)** Resting mast cells. **(F)** Forest plot shows a multivariate Cox analysis of these immune cells. **(G)** K–M curves for the OS of STS in the low- and high-TME score subgroups. **(H)** GSEA identifies the phenotype differences between the TME score high and TME score low subgroups. **(I)** Correlation analysis shows the relationship between the GPR and TME score components. **(J)** K–M curve for the OS of STS in the GRP_low+TME_high, GPR_low+TME_low, GPR_high+TME_high, and GPR_high+TME_low subgroups. **(K)** ROC curves demonstrate the predictive efficiency of the GPR-TME classifier. **(L)** Time-dependent ROC curves demonstrate the predictive efficiency of the GPR-TME classifier.

For the 22 TME cells by CIBERSORT, we performed the K–M survival analysis in tumor samples, yielding five favorable prognostic cells (CD8 T cells, activated NK cells, monocytes, resting mast cells, and M1 macrophages). Meanwhile, we also utilized the multivariate Cox regression analysis with bootstrap methods based on those five cells to establish a prognostic model, resampling 1,000 times with a multivariate Cox analysis each time, and the ratio of Coef to bootstrap SD values serves as the weight in the corresponding model for improving the stability of the prognostic model (**Figures 9A–F**).

According to the above prognostic signatures and calculation formula, we subsequently determined the GPR score and TME score, respectively. According to the median value of the GPR score and TME score in the dataset, tumors were classified into two subgroups (**Figures 7E**, **9G**). Notably, we observed that the low GPR score and high TME score showed a statistically longer survival compared to patients with a high GPR score and low TME score. Tumors with a high GPR score were significantly enriched for ribosome, and those with a low GPR score were enriched for allograft rejection, antigen processing and presentation, asthma, autoimmune thyroid disease, cell adhesion molecules, chemokine signaling pathway, graft-versus-host disease, hematopoietic cell lineage, and type I diabetes mellitus (**Figure 7F**). Similarly, tumors with a high TME score were considerably enriched for asthma, autoimmune thyroid disease, calcium signaling pathway, drug metabolism cytochrome P450, histidine metabolism, tryptophan metabolism, and vascular smooth muscle contraction, whereas tumors with a low TME score were enriched for basal cell carcinoma, ribosome, and spliceosome (**Figure 9H**). Additionally, we performed a correlation analysis among GPR-TME factors (GPR-related genes and TME cells); a correlation coefficient heatmap is presented in **Figure 9I**.

### 3.7 Prognostic value of the established GPR-TME classifier

Based on the above results, we asked whether it would be possible to combine the GPR score and TME score to characterize the GPR TME. Therefore, we combined the GPR score with the TME score and developed the GPR-TME classifier, which resulted in dividing patients into four subgroups: GPR^low^/TME^high^, GPR^low^/TME^low^, GPR^high^/TME^high^, and GPR^high^/TME^low^ (**Figure 9J**). We identified that the GPR-TME classifier presented a statistically different prognosis in STS tumor patients, which demonstrated that both the GPR score and TME score contribute significantly to the prognostic value. Patients from the GPR-TME classifier subgroup were revealed to have the best prognosis compared to patients in the other subgroups. Meanwhile, we combined the GPR^low^/TME^low^ and GPR^high^/TME^high^ subgroups to become the mixed subgroup, because the prognosis of patients in the two subgroups were less divergent. The GPR-TME classifier could significantly distinguish the OS of the patients with STS, and the AUROC of the GPR^low^/TME^high^ subgroup compared to GPR^high^/TME^low^ was 0.617 (**Figure 9K**). The GPR-TME classifier could predict OS at the 3-, 5-, and 7-year survival with AUCs of 0.688, 0.638, and 0.621, respectively (**Figure 9L**).

**Figure 10:**
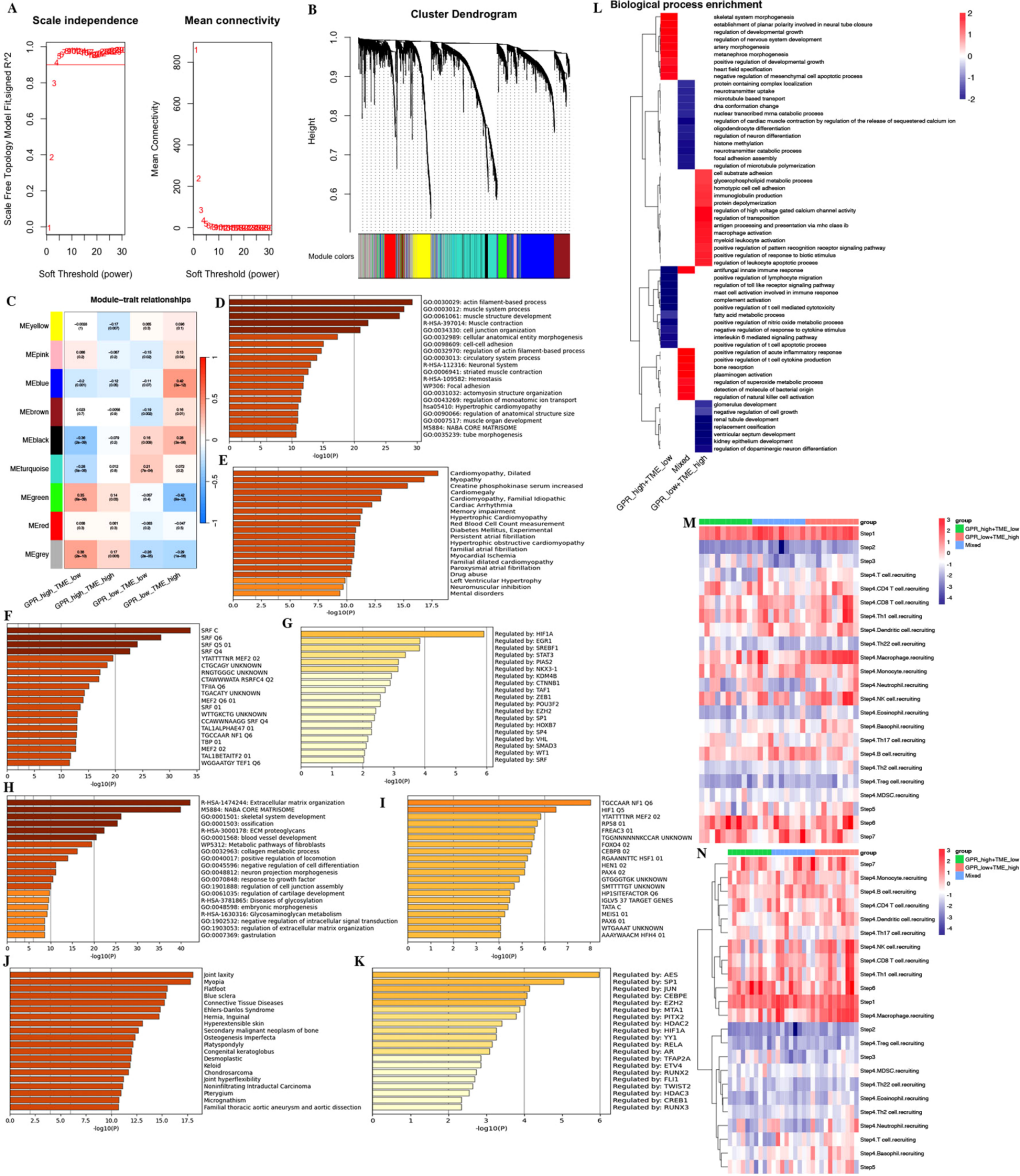
WGCNA and functional enrichment analysis of the GPR-TME classifier. **(A)** Soft threshold of scale free topology model. **(B)** Cluster dendrogram of all genes. **(C)** Gene modules derived from WGCNA show the different clusters among four subgroups. **(D–G)** Enrichment analysis for the GRP_low+TME_high subgroup by Metascape. **(D)** Top 20 annotations. **(E)** Enrichment analysis result in DisGeNET. **(F)** Enrichment analysis result in transcription factor targets. **(G)** Enrichment analysis result in TRRUST. **(H–K)** Enrichment analysis for the GPR_high+TME_low subgroup by Metascape. **(H)** Top 20 annotations. **(I)** Enrichment analysis result in DisGeNET. **(J)** Enrichment analysis result in transcription factor targets. **(K)** Enrichment analysis result in TRRUST. **(L)** Fast gene set enrichment analysis (FGSEA) of the GPR-TME classifier. **(M, N)** Heatmaps for steps 1–7 of TIP analysis based on the GPR-TME classifier.

Furthermore, we performed WGCNA and FGSEA for the molecular signaling pathways based on GPR-TME subgroups according to the significant prognostic differences in the prognostic classifier. WGCNA indicated that the MEgreen module was positively correlated with GPR^high^/TME^low^ and the MEblue module was positively correlated with the GPR^low^/TME^high^ subgroup (**Figures 10A–C**). Metascape results of these two module genes are shown in **Figures 10D–K** (**Supplementary Table S10**). FGSEA identified that the GPR^low^/TME^high^ subgroup was mainly enriched in cell substrate adhesion, glycerophospholipid metabolic process, homotypic cell–cell adhesion, immunoglobulin production, protein depolymerization, regulation of high voltage gated calcium channel activity, regulation of transposition, antigen processing and presentation via major histocompatibility complex (MHC) class Ib, macrophage activation, myeloid leukocyte activation, positive regulation of pattern recognition receptor signaling pathway, positive regulation of response to biotic stimulus, and regulation of leukocyte apoptotic process (**Figure 10L**). The above furthermore demonstrated the synergistic influence of GPR and TME on antigen processing and presentation and immune activation, which implies the significance of an integrated analysis of GPR-TME.

### 3.8 Differences in anti-cancer immunity cycle and immunotherapy response among different GPR-TME subgroups

In the study, we performed TIP analysis for assessing the activity of each step in the anti-cancer immune cycle, which might have a more comprehensive understanding of the anti-cancer role of immune cells that improves immunotherapy guidance (**Figures 10M, N**). We observed that there were differences in steps 1, 4, 6, and 7 of the anti-cancer immune cycle among different GPR-TME subgroups. The GPR^low^/TME^high^ subgroup demonstrated stronger activity in the release of cancer cell antigens (step 1), trafficking of immune cells to tumors (step 4), recognition of cancer cells by T cells (step 6), and killing of cancer cells (step 7). The results were refined to analyze the recruitment of different immune cells in step 4 by the GPR-TME subgroups, which revealed that the GPR^low^/TME^high^ subgroup had a greater ability to recruit immune cells, especially CD8+ T cells, dendritic cells, macrophages, NK cells, monocytes, and Th 1 cells, which might have greater anti-cancer activity in the cycle of immune cell functioning.

### 3.9 Association between the GPR-TME classifier and clinical traits

**Figure 11:**
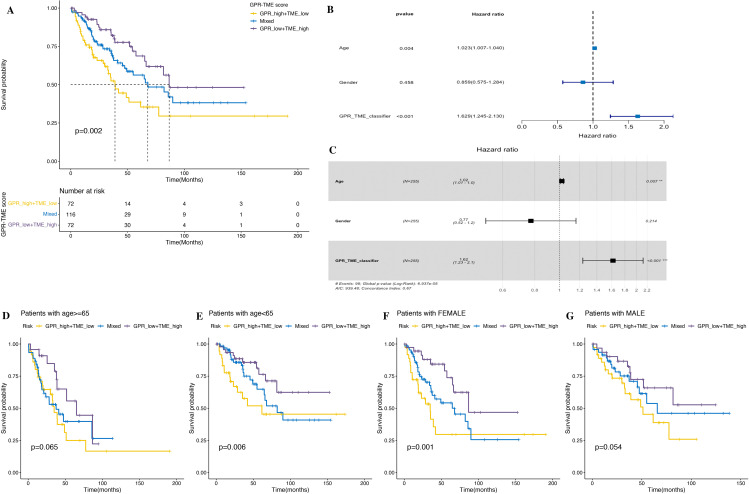
Clinical trait analysis and application of the GPR-TME classifier. **(A)** K–M survival analysis was performed in the GRP_low+TME_high, Mixed, and GPR_high+TME_low groups. **(B, C)** Univariate and multivariate Cox analysis of multiple clinical traits. **(D–G)** K–M curves for the simplified GPR-TME classifier present statistically significant discriminations regardless of age and sex.

Considering the relationship between clinical traits and prognosis, univariate and multivariate Log rank survival analysis and cox regression analysis were conducted. The result of survival analysis found that GPR-high plus TME-low group showed a worse prognosis (**Figure 11A**). The results of univariate Cox regression analysis showed that the age and GPR-TME classifier of patients with STS are risk factors [univariate Cox: age (HR), 1.023; 95% (CI): 1.007–1.04, *p*< 0.01; GPR-TME classifier (HR), 1.629; 95% (CI): 1.245–2.13, *p*< 0.001] (**Figure 11B**). The multivariate Cox regression analysis showed that the age and GPR-TME classifier of patients with STS are risk factors and independent factors for the prognosis of patients with STS [multivariate Cox: age (HR), 1.02; 95% (CI): 1.01–1.00, *p*< 0.01; GPR-TME classifier (HR), 1.62; 95% (CI): 1.23–2.10, *p*< 0.001] (**Figure 11C**). Notably, our analysis furthermore demonstrated that the GPR-TME classifier allowed a further subdivision of patients among young (<65 years) women (**Figures 11D–G**).

### 3.10 Differential patterns of tumor somatic mutations in patients among GPR-TME subgroups

**Figure 12:**
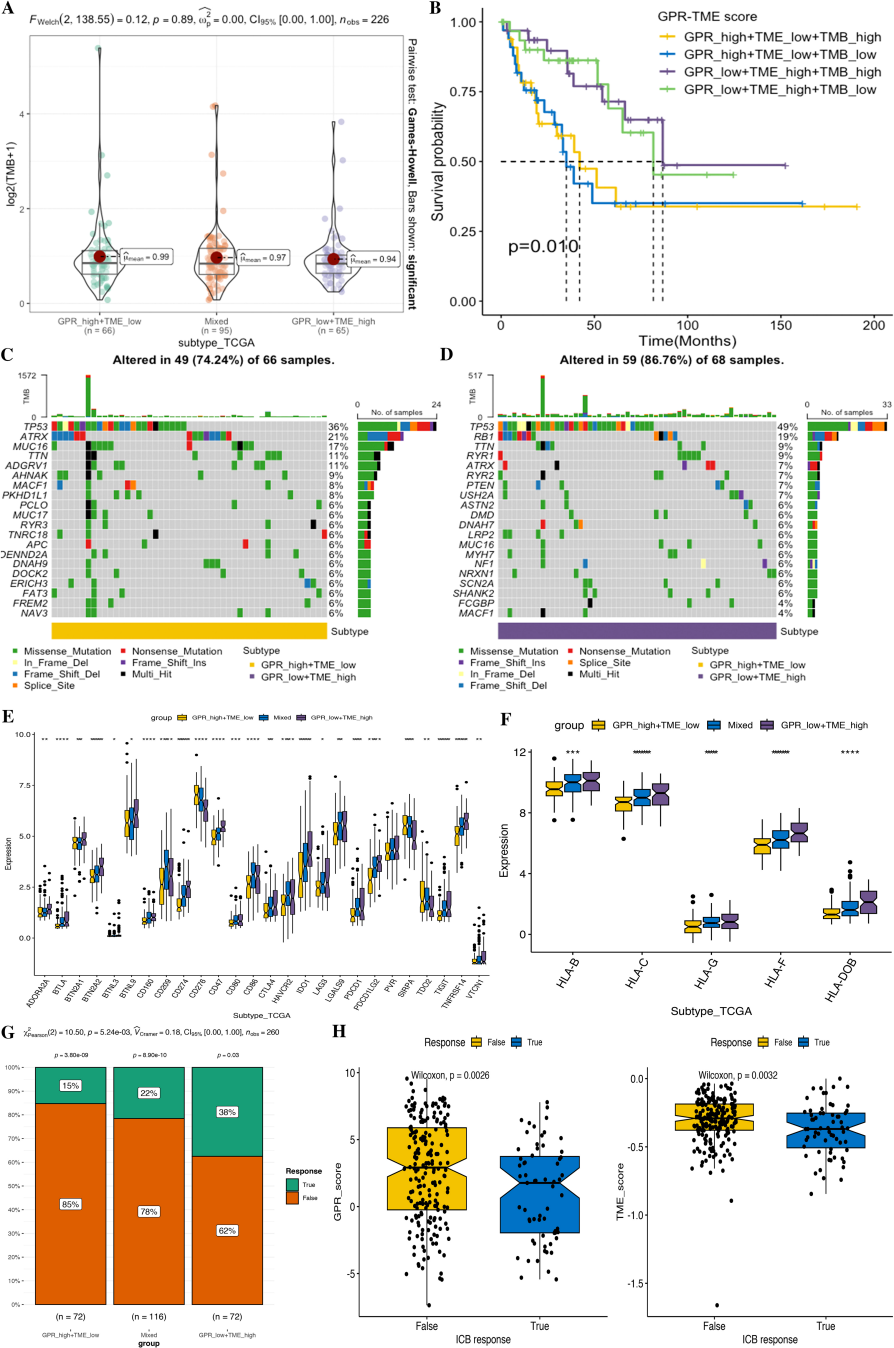
Comparison of tumor somatic mutations, immune-related markers, and therapy response prediction based on the GPR-TME classifier. **(A)** Comparison of TMB level among defined subgroups according to the classifier. **(B)** K–M curves divided by TMB and the GPR-TME classifier of STS. **(C)** Top 20 mutation genes in the GPR_high+TME_low subgroup. **(D)** Top 20 mutation genes in the GPR_low+TME_high subgroups. **(E)** Comparison analysis of ICB responder among subgroups based on the GPR-TME classifier. **(F)** Comparison analysis of the HLA molecules among subgroups based on the GPR-TME classifier. **(G)** Comparison of the different percentages of ICB responder based on the GPR-TME classifier. **(H)** Comparison of GPR scores among patients with different ICB immunotherapy response status.

We next investigated the tumor somatic mutation alterations among different GPR-TME subgroups and presented the top 20 genes with the highest mutation rate in the waterfall map; the GPR^low^/TME^high^ subgroup had a higher mutation rate (**Figures 12A, C, D**). Meanwhile, we also attempted to determine TMB differences among different GPR-TME subgroups, but no significant difference was found; TP53 was the most frequently mutated gene. As shown in **Figure 12B**, to further investigate the effect of TMB on STS, we divided them into TMB^high^ and TMB^low^ groups according to the median value of TMB calculation results and performed joint survival analysis in the GPR^low^/TME^high^ and GPR^high^/TME^low^ groups. The results showed that the GPR^low^/TME^high^/TMB^high^ group had a better prognosis, while the GPR^high^/TME^low^/TMB^low^ group had the worst prognosis, and these results provided additional evidence supporting the notion that individuals in the high-TMB group, specifically the GPR^low^/TME^high^ subgroup, may have a higher likelihood of experiencing an immune response rate. However, TMB status could successfully optimize the predictive efficacy of the GPR-TME classifier. These results might indicate that the GPR-TME classifier is more sensitive than the TMB score to distinguish patients.

### 3.11 Distinct immune response profile in tumors among GPR-TME subgroups

Higher expression of immune checkpoint is associated with a better response to ICI therapy. Thus, we then further investigated the immune response-associated genes among the GPR-TME classifier subgroups, including the expression levels of MHCs and ICIs. It was noted that all MHCs were significantly highly expressed in the GPR^low^/TME^high^ subgroup as shown in **Figures 12E, F**, and most ICIs except CD276, SIRPA, and TDO2 were highly expressed in the GPR^low^/TME^high^ subgroup.

### 3.12 Prediction of therapy response based on the GPR-TME classifier

Furthermore, we tested whether the GPR-TME classifier could be used to predict clinical response in patients who may benefit from ICIs using the TIDE online tool. Then, we evaluated the predictive ability of the GPR-TME classifier in the immunotherapy response from TIDE; the GPR^low^/TME^high^ subgroup had the highest percentage (38%) of patients with immunotherapy response among all subgroups, and the immunotherapy responder of STS presented a significantly lower GPR score but no significant difference in the TME score between the two groups, indicating that the GPR score can independently affect the outcome of immunotherapy (**Figures 12G, H**). Additionally, the Proteomap was used to intuitively reveal the potential mechanism of the GPR-TME classifier predicting therapy response in patients undergoing immunotherapy. Interestingly, the pattern of Proteomap in the GPR^low^/TME^high^ subgroup and in the immunotherapy responder is quite similar, and a higher similarity between the GPR^high^/TME^low^ subgroup and the immunotherapy non-responder was observed (**Supplementary Figure S1**). To summarize, these results might suggest that the pretreatment GPR-TME signature can describe the TME, thus benefiting the prediction of patient’s therapy responses. Similar patterns of Proteomaps are observed in the GPR^low^/TME^high^ subgroup and the ICB responder. These findings may suggest that the pretreatment GPR-TME classifier can depict the tumor immune microenvironment, thereby enhancing the STS patient’s therapy response prediction.

## 4 Discussion

STS is a highly heterogeneous tumor characterized by early invasion, metastasis, resistance to immunotherapy, and poor prognosis, necessitating early diagnosis and tailored treatment strategies (23). GPRs have recently gained more recognition in tumorigenesis, development, and treatment and are an important target for drug development, several of which have been approved for marketing or under development (8, 24). Previous studies suggested that GPRs play a crucial role in the regulation of cancer cells by activating downstream signaling pathways and networks, including metabolism, migration, growth, apoptosis, and cell-specific activities, and may become valuable biomarkers for tumors (12, 25). Meanwhile, TME also plays an important role in cancer biology and tumor prognosis, which may be a promising therapeutic strategy by targeting TME in cancer treatment (26, 27). Recent studies on GPRs and TME strengthen our understanding of their importance in the prognosis and therapy of patients with cancer (10, 18, 28, 29). However, multi-omics application for molecular signatures in STS remains few, which has been confirmed to be a widely useful tool in various cancer research studies, identifying several molecular biomarkers for personalized diagnosis and medicine. In the study, we performed multi-omics approaches to construct a novel GPR signature for early diagnosis, predict prognosis and immunotherapy response for STS, and uncover the underlying molecular mechanisms in the context of PPPM.

Firstly, we developed a novel diagnosis computational framework incorporating 12 MLs and their 127 combinations. Our analysis resulted in the identification of GPRS based on seven GPRs (NCKAP1L, ARHGAP4, ASS1, CD163, SLCO2B1, ALOX5, and ADCY7) to predict the occurrence of STS, which exhibits higher diagnosis accuracy and better clinical translational implications. Secondly, this study firstly conducted a comprehensive bioinformatics analysis of GPRs integrated with TME in STS, resulting in the identification of GPR-TME prognosis classifiers for STS. A more stable prognostic risk assessment model was established using self-service internal validation methods, including seven GPRs (CPXM1, CD48, GPR132, PRF1, CTSW, TBC1D10C, and PLEKHO2) combined with five protective immune cells (CD8 T cells, activated NK cells, monocytes, resting mast cells, and M1 macrophages), to evaluate the prognosis of patients with STS. Meanwhile, we used the novel prognosis classifier for predicting immunotherapy response for STS. The novel classifier exhibits a good predictive ability, clinical prognosis improvement, and clinical translational implications in PPPM framework. These findings provide rational guidance for administering effective personalized immunotherapy in clinical practice. Furthermore, we performed an integrated multi-omics analysis, including scRNA-seq analysis, bulk RNA-seq analysis, and genome analysis, for a deeper understanding of the GPRs combined with TME in STS. We identified the associations of GPRS and the GPR-TME classifier with the development and prognosis of STS, revealing their biological evidence and the molecular basis and an underlying mechanism across multi-omics levels, providing biological evidence in guiding personalized medicine approaches. Lastly, we performed a novel comprehensive bioinformatics analysis that integrated DEGs, ssGSEA, and WGCNA in a bulk transcriptome level and the AddModuleScore in single-cell transcriptome for identifying GPRSs. The findings in the study provided new insights for GPR in STS by uncovering GPRSs and providing potential diagnosis and therapy targets in GPRs for STS.

In the study, we developed a novel computational framework to identify a stable and reliable diagnosis for GPRS. We incorporated 12 machine learnings with their 127 combinations in a training set, and subsequently replicated them in the training, test, meta, and GSE17674 sets. Based on the framework, we successfully fitted a consensus diagnosis model with high accuracy and translatability, and the Stepglm[both]+Enet[alpha=0.6] algorithm was selected as the final model with its high average accuracy, low model gene number, and optimal model performance power. The combined application of multiple machine learning algorithms enables more efficient variable dimensionality reduction, thus facilitating the development of accurate and simple predictive models. Model performance was assessed using ROC curves, C indexes, and calibration curves in the validation datasets. All evaluation methods demonstrated the excellent diagnostic performance of our GPRS models in predicting the onset of STS, which can be employed in high-risk individuals with developing STS and for the early intervention and appropriate treatment of such individuals.

To provide a convenient tool for diagnosing the occurrence of STS, we constructed a nomogram that integrated seven GPR-related genes (NCKAP1L, ARHGAP4, ASS1, CD163, SLCO2B1, ALOX5, and ADCY7). The nomogram demonstrated satisfactory discrimination, with the ROC curve and C index reflecting its high predictive accuracy. The calibration curve further confirmed the accuracy of the nomogram by showing close agreement between predicted and observed diagnostic rates. The diagnostic model was established by machine learning to predict the occurrence of STS; the diagnostic tool for determining the occurrence of STS has a good predictive ability. Early diagnosis of STS may improve prognosis and clinical outcomes. These findings hold significant and powerful implications for the early diagnosis and prompt treatment of GPRS in STS.

Nck-associated protein 1 (NCKAP1) as part of the WAVE (WASP-family verprolin-homologous protein) complex plays an essential role in disease pathogenesis and poor prognosis in several cancers by regulating various intracellular processes through apoptosis, migration, and invasion (30, 31). Nck-associated protein 1-like (NCKAP1L) is a hematopoietic lineage-specific regulator of the Nap1l subunit of the WAVE complex, which signals downstream of activated Rac to stimulate F-actin polymerization in response to engagement of various immune receptors, and NCKAP1L defects would lead to a novel syndrome combining immunodeficiency, lymphoproliferation, and hyperinflammation (32). ARHGAP4 is a novel negative regulator of Rho GTPas‐activating protein (GAP) family proteins inhibiting axon outgrowth and cell motility, and a novel regulator of HDAC2/β-catenin pathway with a critical role in tumorigenesis. Proteins encoded by ARHGAP4 can regulate the binding between GTPase and rat sarcoma (RAS) family members, whose negative regulation involves the small G protein of the Rho family and associated with tumorigenesis in various cancers, including head and neck squamous cell carcinoma, glioblastoma, breast, lung, pancreatic, liver, colon, and prostate cancers (33, 34). Furthermore, ARHGAP4 is associated with immune cells (B, CD8+ and CD4+ T, macrophages, neutrophils, and dendritic cells) and may be a potential biomarker for the prognosis of CRC (34). A previous study suggested that ASS1 plays a critical role in controlling the activation of inflammatory macrophage and in antibacterial defense by depletion of cellular citrulline, which is an innate immune-signaling metabolite that engages a metabolic checkpoint for proinflammatory responses (35). Meanwhile, the somatic silence or downregulation of ASS1 is very common in various cancers, and ASS1 might be a tumor suppressor in breast cancer (36). CD163 as a marker of M2 macrophage might contribute to predict aggressiveness and prognosis of Kazakh ESCC with the increased number of M2 macrophages (37), and CD163 also contributes to gliomagenesis via casein kinase 2 that might serve as a therapeutic target for glioma (38). A previous study suggested that SLCO2B1 as a heme transporter is enriched in microglia in the brain and required for heme analog import, which enhances cellular iron availability (39). ALOX5 plays a critical role in cell death by ways of inflammation and lipid peroxidation including apoptosis, pyroptosis, and ferroptosis, whose activity is regulated by several factors including protein phosphorylation, ALOX5-interacting protein, redox state, and metal ions (40). ALOX5 exhibits antitumor and drug-sensitizing effects and has a therapeutic potential in mixed lineage leukemia (MLL)-rearranged leukemia (41). Meanwhile, ALOX5 may be a valuable therapeutic target and prognostic biomarker for bladder cancer, in which the deficiency of ALOX5 might contribute to BCa progression by mediating ferroptosis escape (42). Adenylate cyclase 7 (ADCY7) plays a critical role in nervous system diseases, inflammatory responses, and immune responses, and ADCY7 was abnormally expressed in multiple cancers and may be a prognostic biomarker of tumorigenesis (43). A previous study showed that ADCY7 deficiency resulted in decreased cell growth, elevated apoptosis, and lower c-Myc expression of these leukemia cells, indicating that GPR signaling contributes to AML pathogenesis and that ADCY7 supports the development of AML; the inhibition of ADCY7 may be a novel treatment strategy for AML (44).

When considering targeting GPR combined with immunotherapy for the treatment of STS, the signatures based on the combination of GPR and TME might enable both clinical classification and optimizing therapy strategies. In our study, we systematically utilized large-scale STS sets to assess the integrated value of GPR TME for prognostic and immunotherapy response based on the GPR-TME classifier. In this study, we investigated the joint effect of GPR and TME on STS, first using differential expression analysis, univariate Cox regression analysis, LASSO algorithm, and multivariate Cox regression analysis, and identified a total of seven genes (CPXM1, CD48, GPR132, PRF1, CTSW, TBC1D10C, and PLEKHO2) related to STS prognosis and constructed a seven-GPR risk signature. The TME score was further calculated based on the five protective immune cells (CD8 T cells, activated NK cells, monocytes, resting mast cells, and M1 macrophages). Finally, a GPR-TME classifier that can predict patient prognosis and assist in subsequent immunotherapy analysis was established by the combination of the above two risk profiles, and its value in terms of the prognosis and immunotherapy in patients with STS was assessed. We identified that the GPR score was a risk factor for STS prognosis, while the TME score was a protective factor. Upon simplification, the GPR-TME classifier was identified as an independent prognostic factor for patients with STS, and the GPR^low^/TME^high^ subgroup has the best prognosis outcome and clinical immunotherapy. This classifier’s prognosis predictive value was independent of the patient’s age and sex, indicating a stable prediction efficiency and the robustness of the classifier. The time-dependent ROC curves confirmed the sensibility and specificity of this risk signature. The model was also used for single-cell RNA statistical processing, cell–cell communications, tumor mutational load, and immunotherapy analysis.

CPXM1 is an epigenetic factor involved in many physiological processes including osteoclast differentiation and adipogenesis, which might be a novel biomarker for the detection and treatment of various cancers, including gastric cancer, ovarian cancer, breast cancer, neck squamous cell carcinoma, myelodysplastic syndrome, and papillary thyroid (45). CD48 may play an important role in mediating the immune response in both immune activation and suppression, which binds to CD2 and is involved in a wide variety of innate and adaptive immune responses, and CD48 interaction with its high-affinity receptor 2B4 (CD244) leads to monocyte/macrophage-elicited NK cell dysfunction in HCC (46). CD48 is a key molecule of immunomodulation affecting prognosis in glioma, and combining CD48 blockade with PD-L1 may be a promising immunotherapy approach for specific subpopulations of glioma (47). A previous study suggested that the lactate–Gpr132 axis is a driver of breast cancer metastasis by stimulating tumor–macrophage interplay and revealed potential new therapeutic targets for breast cancer treatment (48). The pore-forming protein perforin (PRF1) is a definite marker of the killing ability of immune cells and is involved in the establishment of immune homeostasis, elimination of pathogens, and tumor surveillance. PRF1 might be related to better survival in multiple cancers, including melanoma, bladder cancer, head and neck squamous cell carcinoma, and ovarian cancer (49). A previous study suggested that CTSW inhibits IL-2R signaling in pTreg cells by cytosolic interaction with and processing of CD25, repressing signal transducer and activator of transcription 5 activation to restrain pTreg cell generation and maintenance (50). Cancer immunotherapy approaches target signaling pathways that are highly synonymous between CD4 and CD8 T-cell subsets and, therefore, often stimulate nonspecific lymphocyte activation, resulting in cytotoxicity to otherwise healthy tissue. TBC1D10C is a selective, constitutive suppressor of the CD8 T-cell antitumor response, and the Tbc1d10c–Map3k3–NF-κB signaling axis is a viable therapeutic target to promote CD8 T-cell antitumor immunity while circumventing CD4 T cell-associated cytotoxicity and NF-κB activation in tumor cells (51). PLEKHO2 is a novel inhibitor of apoptosis and necroptosis, and plays a key role in regulating RIPK1 ubiquitination and activation, which inhibits TNFα-induced cell death by suppressing RIPK1 activation (52, 53).

We performed a systematic exploration of the functions and pathways involved in different subgroups for providing a deeper understanding into the transcriptional regulation mechanisms of the GPR-TME classifier in STS. The functional enrichment revealed that the GPR-TME classifier was associated with tumor growth, and there is potential influence on cellular communication in STS. In addition, by conducting immune function and antitumor immune cycle analyses of TIP, we found that the GPR^low^/TME^high^ subgroup had a stronger inflammation-promoting, cytolytic activity, and T-cell co-inhibition activity and was more active in most of the anti-cancer immune cycle steps. This further revealed that the GPR^low^/TME^high^ subgroup has stronger antitumor immune activity, which is corroborated by the results obtained in the functional enrichment analysis. Moreover, multiple algorithm methods for functional annotation revealed different biological process enrichments among GPR-TME subgroups. This may imply that the host tumor profiles of the various GPR-TME subgroups share certain common characteristics.

In scRNA-seq analysis, we observed a significantly higher GPR activity in B cells, macrophages, NK cells, CD4+ T cells, CD8+ T cells, and T cells, and GPRs genes may play a role in immune cell function (54–56). We found that B, NK, and macrophage cells in both GPR-high and GPR-low risk groups and CD4+ and CD8+ T cells in the GPR-low risk group interacting with MIF-(CD74 + CXCR4) ligand–receptor relationships were extremely correlated, which indicated that the interactions of those cells with other cell types are related to MIF signaling pathway (57, 58). These findings suggest that high GPR activity enhances the cellular communication function of macrophage cells while suppressing the cellular communication function of B cells, CD4 + T cells, CD8 + T cells, NK cells, and mastocyte cells. The above results might elucidate the mechanism behind the GPR-TME classifier predicting prognosis and therapy responses.

Furthermore, immune checkpoints and tumor antigen presentation play a key role in tumor therapy (59), and an intriguing result showed that both activating and inhibitory immune markers were, in general, highly expressed in the GPR^low^/TME^high^ subgroup, suggesting that a stronger antitumor immune response would likely be restored through immune checkpoint blockade in the GPR^low^/TME^high^ subgroup. Moreover, somatic mutation results provide additional evidence supporting the notion that individuals in the high-TMB group, specifically the GPR^low^/TME^high^ subgroup, may have a higher likelihood of experiencing an immune response rate. Finally, we investigated whether the GPR-TME signatures could predict the response to immunotherapy in patients with STS, and the response to immunotherapy differed among risk groups in this model, with lower GPR signatures or higher TME signatures implying better levels of immune response. We applied the model to the ICI treatment cohort and found that the model could be used to predict the efficacy of ICI treatment in patients with STS. This implies that the GPR-TME classifier is of great research value in immunotherapy and may be applied for the pre-immunotherapy stratification of patients with STS. In our GPR-TME classifier, 38% of patients in the GPR^low^/TME^high^ subgroup were successfully predicted to benefit from immunotherapy regardless of the molecular subtyping. The similarity of Proteomap patterns between the GPR^low^/TME^high^ subgroup and TIDE immunotherapy-responder subgroups might reveal a certain commonality in a determinative interplay between patients’ immune system and cancer cells, which further indicated the therapeutic predictive value of the GPR-TME classifier. The findings provide an explanation from a multi-omics perspective on the better prognosis and better response to immunotherapy observed in the GPR^low^/TME^high^ subgroup. In summary, this study based on integrated multi-omics bioinformatics analysis provides some new biomarkers of GPRs for the diagnosis and prognosis of STS and may aid to develop new precise and effective targeted drugs and prevent the progression of STS in the context of PPPM. The results of our study provide new ideas for prognosis prediction as well as treatment of STS, which may enable the clinical classification and optimization of therapy strategies.

Meanwhile, some limitations should still be addressed, even though we innovatively integrated GPR and TME for predicting prognosis and immunotherapy response in STS with the bootstrap method to enhance our model’s stability and reliability in the study. First, the reliance on public datasets may constrain the generalizability of the classifier. Despite internal and external validation, further evaluation using large-scale, prospective, multi-center cohorts is essential. Second, the GPRS and GPR-TME classifier were all based on gene expression that additional *in vivo* and *in vitro* experimental studies require for further validation and unraveling the intricate molecular pathways involved. Lastly, although we had predicted the immunotherapy response of the classifier, large-scale prospective clinical and real-world data are still needed to further confirm its clinical applications in personalized medicine selection for clinicians.

## 5 Conclusion

In this study, we firstly constructed a GPRS that can serve as a promising tool for diagnosis and prognosis prediction, targeted prevention, and personalized medicine in patients with STS. Incorporating GPR-combined cellular landscape into the PPPM framework will provide a unique opportunity for clinical management and precise personal treatment. Additionally, we provided novel insights into the molecular mechanisms underlying the occurrence, development, and progression of STS from a multi-omics perspective.

## Data Availability

The original contributions presented in the study are included in the article/**Supplementary Material**, further inquiries can be directed to the corresponding author/s.

## Supplementary material

Supplementary Table S1 Key module genes related to GPR in bulk RNA-seq.

Supplementary Table S2 KEGG of key module genes.

Supplementary Table S3 BP of key module genes.

Supplementary Table S4 CC of key module genes.

Supplementary Table S5 MF of key module genes.

Supplementary Table S6 Identification of 151 GPR-related genes (GPR genes).

Supplementary Table S7 AUROCs across all training and validation datasets of all machine learning combinations.

Supplementary Table S8 Features across all training and validation datasets of all machine learning combinations.

Supplementary Table S9 Potential prognostic genes identified by univariate Cox and Lasso regression analysis.

Supplementary Table S10 Key module genes related to the GPR-TME classifier.

Supplementary Figure 1 Functional analysis by Proteomaps in GPR_high+TME_low **(A)**, up genes; **(B)**, down genes and responder **(C)**, up genes; **(D)**, down genes) of STS under ICB immunotherapy.
