# Supplementary figures and images for: Integrated multi-omics analysis and machine learning identify G protein-coupled receptor-related signatures for diagnosis and clinical benefits in soft tissue sarcoma
Source: Front Immunol. 2025 Jul 21;16:1561227. doi: 10.3389/fimmu.2025.1561227 (PMC12318986; doi:10.3389/fimmu.2025.1561227)

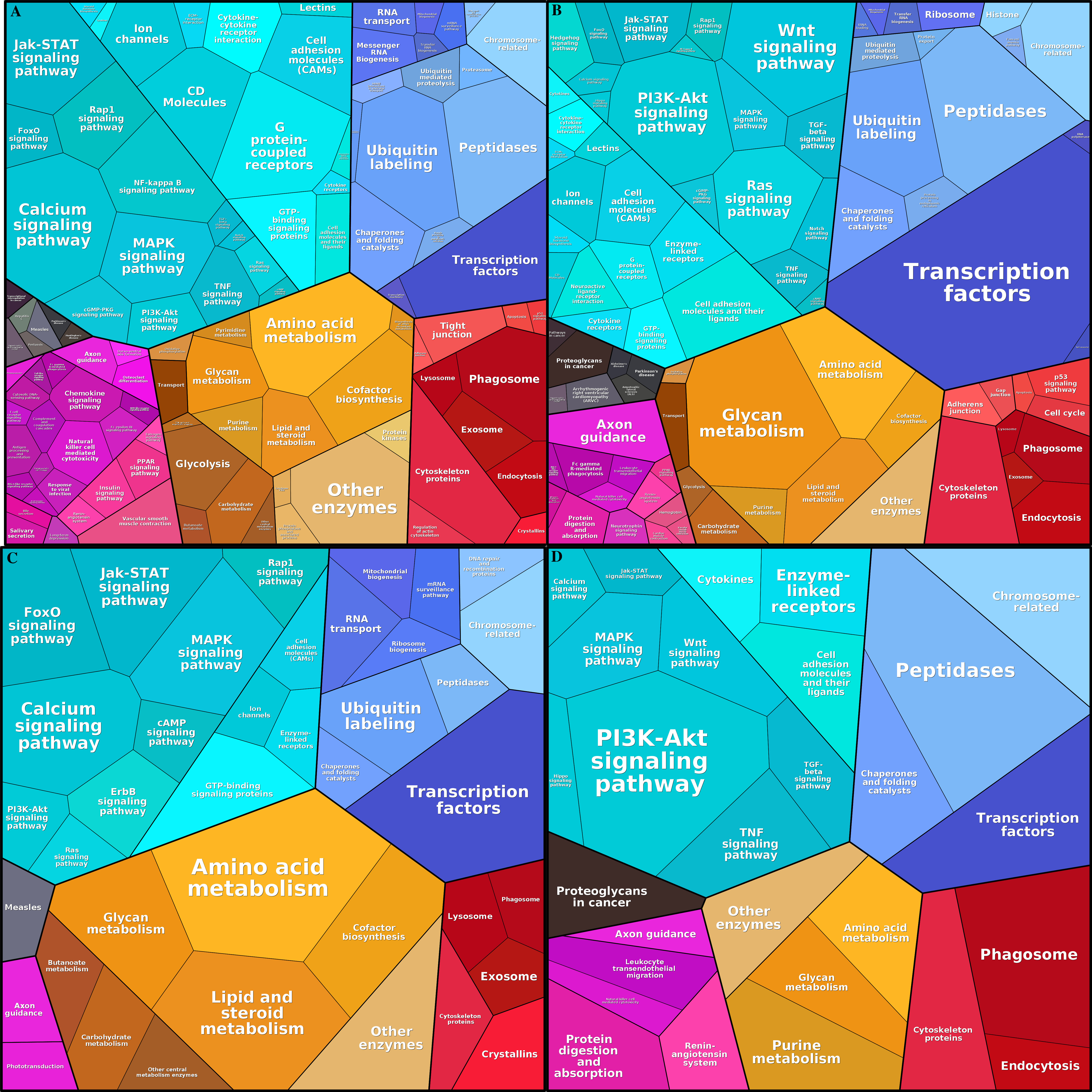

Supplement: Supplementary file 2 [file Image1.jpeg]
